# A wideband, high-resolution vector spectrum analyzer for integrated photonics

**DOI:** 10.1038/s41377-024-01435-z

**Published:** 2024-04-08

**Authors:** Yi-Han Luo, Baoqi Shi, Wei Sun, Ruiyang Chen, Sanli Huang, Zhongkai Wang, Jinbao Long, Chen Shen, Zhichao Ye, Hairun Guo, Junqiu Liu

**Affiliations:** 1 International Quantum Academy, 518048 Shenzhen, China; 2 Shenzhen Institute for Quantum Science and Engineering, Southern University of Science and Technology, 518055 Shenzhen, China; 3 Department of Optics and Optical Engineering, University of Science and Technology of China, 230026 Hefei, China; 4 Hefei National Laboratory, University of Science and Technology of China, 230088 Hefei, China; 5 Qaleido Photonics, 518048 Shenzhen, China; 6 Key Laboratory of Specialty Fiber Optics and Optical Access Networks, Shanghai University, 200444 Shanghai, China

**Keywords:** Microresonators, Silicon photonics, Infrared spectroscopy, Integrated optics

## Abstract

The analysis of optical spectra—emission or absorption—has been arguably the most powerful approach for discovering and understanding matter. The invention and development of many kinds of spectrometers have equipped us with versatile yet ultra-sensitive diagnostic tools for trace gas detection, isotope analysis, and resolving hyperfine structures of atoms and molecules. With proliferating data and information, urgent and demanding requirements have been placed today on spectrum analysis with ever-increasing spectral bandwidth and frequency resolution. These requirements are especially stringent for broadband laser sources that carry massive information and for dispersive devices used in information processing systems. In addition, spectrum analyzers are expected to probe the device’s phase response where extra information is encoded. Here we demonstrate a novel vector spectrum analyzer (VSA) that is capable of characterizing passive devices and active laser sources in one setup. Such a dual-mode VSA can measure loss, phase response, and dispersion properties of passive devices. It also can coherently map a broadband laser spectrum into the RF domain. The VSA features a bandwidth of 55.1 THz (1260–1640 nm), a frequency resolution of 471 kHz, and a dynamic range of 56 dB. Meanwhile, our fiber-based VSA is compact and robust. It requires neither high-speed modulators and photodetectors nor any active feedback control. Finally, we employ our VSA for applications including characterization of integrated dispersive waveguides, mapping frequency comb spectra, and coherent light detection and ranging (LiDAR). Our VSA presents an innovative approach for device analysis and laser spectroscopy, and can play a critical role in future photonic systems and applications for sensing, communication, imaging, and quantum information processing.

## Introduction

The analysis of light and its propagation in media is fundamental in our information society. The discovery of light refraction and dispersion in media has resulted in the invention of prisms and gratings that are ubiquitously used in today’s optical systems for imaging, sensing, and communication. Key enabling building blocks to these applications are dispersive elements that separate light components of different colors (i.e. frequencies) either spatially or temporally^1^, with precisely calibrated chromatic dispersion. With these elements, modern optical spectrum analyzers (OSA) and spectrometers can deliver unrivaled frequency resolution, large dynamic range, and wide spectral bandwidth of hundreds of nanometers. Time-stretched systems^2^ can probe ultrafast and rare events in complex nonlinear systems.

For spectrum analysis, precise and broadband frequency-calibration of dispersive elements is pivotal. Due to the ultimate need for spectrometers with reduced size, weight, cost, and power consumption, extensive efforts have been made to create miniaturized spectrometers^3–12^ and broadband laser sources^13–30^ based on integrated waveguides. For these devices, frequency-calibration is particularly crucial yet challenging since the dispersion of integrated waveguides can be significantly altered by the structures and sizes^31,32^. Meanwhile, stationary phase approximation for time-stretch dispersive Fourier transform^33^ necessitates carefully frequency-calibrated elements that are strongly dispersive. For these purposes, optical vector network analyzers (OVNA) are viable tools. Analog to an electrical VNA, an OVNA enables direct characterization of the linear transfer function (LTF) of passive devices, therefore allowing simultaneous measurement of transmission (i.e. loss), phase response, and dispersion^34–36^. Previously demonstrated OVNAs are based on interferometry^35,37^, optical channel estimation^36,38^, single-sideband modulation^39,40^, and frequency-comb-assisted asymmetric double sidebands^41^. Despite, all these methods have limited measurement bandwidth of sub-terahertz to a few terahertz. Therefore for booming demands to understand and to engineer devices used for broadband laser sources that span over tens of terahertz, including optical frequency combs^13–15^, parametric oscillators^16,18,19^, quantum frequency translators^20,21^, supercontinua^22–25^, and parametric amplifiers^26–29^, all these methods fail.

Here we demonstrate a new paradigm of vector spectrum analysis that unites OVNA for passive devices and OSA for active laser sources in one setup. Our vector spectrum analyzer (VSA) can measure LTF and dispersion properties of passive devices, or coherently map an optical spectrum into the RF domain.

## Results

**Fig. 1:**
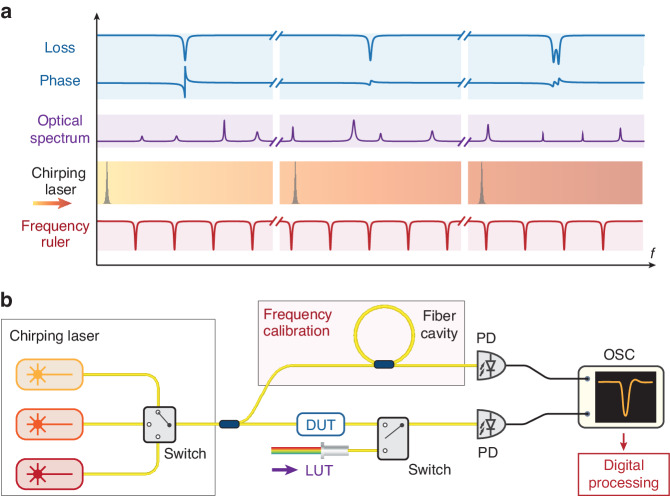
Principle and architecture of the vector spectrum analyzer (VSA). **a** The principle of our VSA is based on a chirping CW laser that is sent to and transmits through a device under test (DUT), or interferes with any light under test (LUT). The DUT can be any passive device, and the LUT can be any broadband laser source. The transmission spectrum of the chirping laser through the DUT, and the beatnote signal generated from the interference between the chirping laser and the LUT, are both time-domain traces. Together with a “frequency ruler” for calibration, the chirping laser coherently maps the time-domain trace into the frequency domain. This trace carries the information of the DUT’s loss, phase and dispersion over the chirp bandwidth (blue). For LUT, the chirping laser beats progressively with different frequency components of the optical spectrum, thus, analyzing the beat signal in the RF domain allows extraction of the spectral information (purple). Critical to this frequency-time mapping is precise and accurate calibration of the instantaneous laser frequency during chirping. This requires referring the chirping laser to the frequency ruler (red). **b** Experimental setup. The chirping laser unit can be a single laser, or multiple lasers that are bandwidth-cascaded. The latter allows extension of the full spectral bandwidth by seamless stitching of individual laser traces into one trace. The chirping laser power is then split into two branches. The upper branch is directed to the frequency-calibration unit (i.e. the “frequency ruler”), which in our case, is a phase-stable fiber cavity of 55.58 MHz FSR. The lower branch is sent to the DUT or the LUT. For the two branches, the photodetector signals are recorded with an oscilloscope and digitally processed offline. PD photodetector, OSC oscilloscope

The principle of our VSA is illustrated in Fig. 1a. A continuous-wave (CW), widely chirping laser, is sent to and transmits through a device under test (DUT), or interferes with a light under test (LUT). During laser chirping, for the DUT, the frequency-dependent LTF containing its loss and phase information is photodetected and recorded. Particularly, the phase is extracted by interfering the light exiting the DUT with a reference branch. For the LUT, the chirping laser beats progressively with different frequency components of the optical spectrum, and the beatnote signal is photodetected, recorded in the RF domain, and further processed with a digital narrow-band-pass filter offline. In either case, the VSA outputs a time-domain trace, with each data point corresponding to the DUT’s instantaneous response, or the LUT’s instantaneous beatnote from interference, at a particular frequency during laser chirping. In short, the chirping laser coherently maps the frequency-domain response into the time domain, which is digitally processed to retrieve the frequency-domain response. Since the laser cannot chirp perfectly linearly, critical to this frequency-time mapping is precise and accurate calibration of the instantaneous laser frequency at any given time. This requires referring the chirping laser to a calibrated “frequency ruler”.

Following this principle, we construct the setup as shown in Fig. 1b. A widely tunable, mode-hop-free, external-cavity diode laser (ECDL, Santec TSL) is used as the chirping laser. Cascading multiple ECDLs covering different spectral ranges allows the extension of full spectral bandwidth, which is 1260–1640 nm (55.1 THz) in our VSA with three ECDLs (see Note 1 in Supplementary Materials).

The ECDL’s CW output is split into two branches. One branch is sent to the DUT or the LUT, selected by an optical switch. The other branch is a frequency-calibration unit based on a fiber cavity. Such frequency calibration involves relative- (i.e. the frequency change relative to the starting laser frequency) and absolute-frequency calibration (i.e. accurately measured starting laser frequency). The absolute-frequency calibration is performed by referring to a built-in wavelength meter with an accuracy of 200 MHz (see Note 1 in Supplementary Materials). The relative-frequency calibration is described in the following.

### Relative frequency calibration of the VSA

**Fig. 2:**
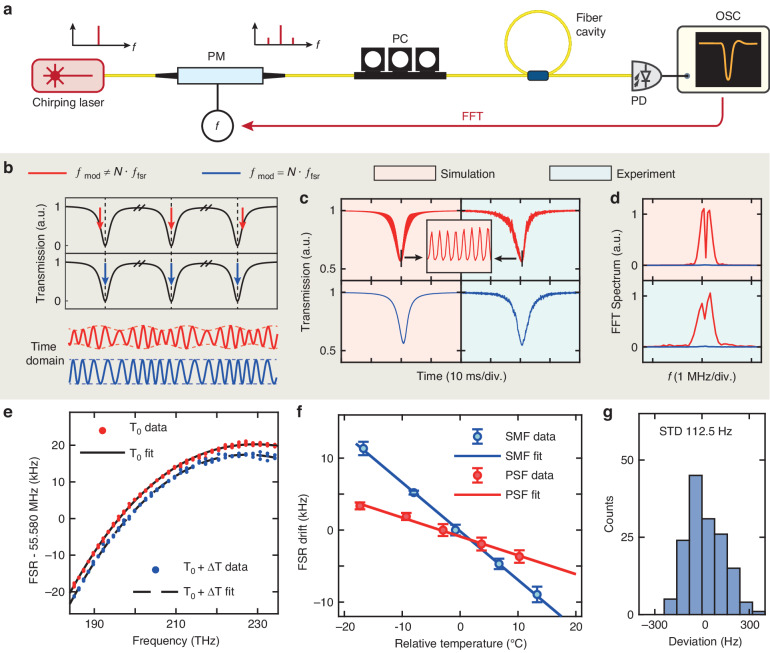
Frequency-calibration of the fiber cavity. **a** Experimental setup. PM phase modulator, PC polarization controller, FFT fast Fourier Transformation. **b–d** Principle of the frequency-calibration process of the fiber cavity’s FSR. Charts compare the differences when ${f}_{\mathrm{mod}}\ne N\cdot {f}_{\text{fsr}}$ (red arrows and curves) and ${f}_{\mathrm{mod}}=N\cdot {f}_{\text{fsr}}$ (blue arrows and curves). The three red/blue arrows in Panel **b** mark the incident CW laser with the two sidebands generated from it. From the experimental data (with blue background) and simulation (with red background), the differences between ${f}_{\mathrm{mod}}\ne N\cdot {f}_{\text{fsr}}$ and ${f}_{\mathrm{mod}}=N\cdot {f}_{\text{fsr}}$ are illustrated by: 1. The envelope modulation on the time-domain trace (Panel **b** bottom); 2. The resonance profile (Panel **c**); 3. The Fourier peaks in the RF domain via FFT (Panel **d**). **e** Measured fiber cavity’s FSR variation over the 55.1 THz frequency range with fitted dispersion. We perform the measurement at two different temperatures ${T}_{0}$ and ${T}_{0}+\Delta T$, where *T*_0_ = 23.5 °C and Δ*T* = 9.3 °C. **f** For fiber cavities made of single-mode fibers (SMF) or phase-stable fibers (PSF), the measured cavity FSR drifts versus relative temperature change, as well as the linear fit. **g** Totally 150 measurements of the fiber cavity’s FSR show a standard deviation (STD) of 112.5 Hz

Figure 2 illustrates the principle of relative-frequency calibration. We use a fiber cavity with an equidistant grid of resonances as the frequency ruler^42,43^. By counting the number of resonances passed by the chirping laser and multiplying the number with the fiber cavity’s free spectral range (FSR, *f*_fsr_), the laser frequency excursion is calculated. Extrapolation of laser frequency between two neighboring fiber cavity’s resonances further improves frequency resolution, precision and accuracy, which will be discussed later. Therefore, critical to this method is the measurement precision of *f*_fsr_ and compensation of fiber dispersion to account *f*_fsr_ variation over the 55.1 THz spectral range.

The experimental setup to calibrate *f*_fsr_ is shown in Fig. 2a. The ECDL’s CW output is phase-modulated by an RF signal generator to create sidebands. Though consisting of multiple CW components, i.e. the carrier and sidebands, the amplitude is constant in the time domain. The carrier and both sidebands are together sent into the fiber cavity with maintained polarization. The transmitted signal through the fiber cavity is probed by a 125-MHz-bandwidth photodetector, recorded by an oscilloscope, digitally processed, and fed back to the RF signal generator. Based on the fiber cavity length, an initial value of the fiber cavity’s FSR, Δ*f*_0_ = 55.58 MHz, is estimated. The RF driving frequency ${f}_{\mathrm{mod}}$ of the phase modulator is set to ${f}_{\mathrm{mod}}=N\cdot \Delta {f}_{0}$, where *N* is an integer ($N=3$ in our case).

Since $\Delta {f}_{0}\ne {f}_{\text{fsr}}$, as shown in Fig. 2b, the carrier and both sidebands are misaligned with the respective three resonances in the frequency domain. In this case, the three CW components experience different cavity responses (including loss and phase). Thus, the superposition of light fields varies accordingly, leading to a beatnote at the output. When ${f}_{\mathrm{mod}}$ is slightly varied such that ${f}_{\mathrm{mod}}=N\cdot {f}_{\text{fsr}}$ is satisfied, the carrier and both sidebands can be simultaneously aligned with the fiber cavity’s resonances, experiencing the same cavity’s response. Thus, the superposition of light fields of these CW components is unaffected at the output, producing a constant current on the photodetector.

Experimentally, the laser chirps across a resonance of the fiber cavity, and the time-domain interference at the fiber cavity’s output is photodetected and recorded by the oscilloscope. The time-domain trace is then normalized to the level of off-resonant signal power, as the transmission spectrum of a resonance shown in Fig. 2c. When ${f}_{\mathrm{mod}}\ne N\cdot {f}_{\text{fsr}}$, the resonance profile is modulated at the frequency ${f}_{\mathrm{mod}}$ (red curves). As ${f}_{\mathrm{mod}}$ approaches $N\cdot {f}_{\text{fsr}}$, i.e., $\left|{f}_{\mathrm{mod}}-N\cdot {f}_{\text{fsr}}\right|\to 0$, the modulation amplitude decreases. When ${f}_{\mathrm{mod}}=N\cdot {f}_{\text{fsr}}$, the resonance profile is unaffected as a normal Lorentzian profile probed by a single CW laser (blue curves). We simulate this modulation behavior (left red panels) which agrees with the experimental data (right blue panels). The modulation amplitude is extracted with fast Fourier transformation (FFT) as shown in Fig. 2d, where red curves represent ${f}_{\mathrm{mod}}\ne N\cdot {f}_{\text{fsr}}$ and blue curves represent ${f}_{\mathrm{mod}}=N\cdot {f}_{\text{fsr}}$. When ${f}_{\mathrm{mod}}\ne N\cdot {f}_{\text{fsr}}$, a binary search to minimize $\left|{f}_{\mathrm{mod}}-N\cdot {f}_{\text{fsr}}\right|$ is performed until the modulation peaks vanish, signaling ${f}_{\mathrm{mod}}=N\cdot {f}_{\text{fsr}}$. For more details on the calibration process and numerical simulations, see Note 2 in Supplementary Materials.

We apply this method to measure the fiber cavity’s ${f}_{\text{fsr}}$ from 1260 to 1640 nm wavelength (55.1 THz frequency range) with an interval of 10 nm, at an ambient temperature of *T*_0_ = 23.5 °C. The fiber cavity is made of phase-stable fibers (PSF, described later). Figure 2e shows that plots and analysis of frequency-dependent ${f}_{\text{fsr}}$ enable extraction of the fiber dispersion using a cubic polynomial fit (see Note 3 in Supplementary Materials). This dispersion-calibrated fiber cavity’s resonance grid is used as the frequency ruler in our VSA and following experiments.

We further characterize the temperature stability of ${f}_{\text{fsr}}$. The fiber cavity is heated and its ${f}_{\text{fsr}}$ shift versus the relative temperature change at 1490 nm is measured, as shown in Fig. 2f. In addition, we compare two types of fibers to construct the cavity: the normal single-mode fiber (SMF, blue data) and phase-stable fiber (PSF, red data). The linear fit shows that the PSF-based fiber cavity features a temperature sensitivity of $\text{d}{f}_{\text{fsr}}/\text{d}T=-262$ Hz/K, in comparison to −676 Hz/K of the SMF. The lower $\text{d}{f}_{\text{fsr}}/\text{d}T$ of PSF is the reason why we use PSF instead of SMF. Correspondingly, 1 K temperature change (the level of our ambient temperature stabilization and control) causes ~240 MHz cumulative error of the PSF-based fiber cavity over the entire 55.1 THz range.

We also measure the fiber cavity’s dispersion at elevated temperature ${T}_{0}+\Delta T$, where Δ*T* = 9.3 °C. Figure 2e shows that, the two measured fiber dispersion curves at different temperatures are nearly identical except with a global offset in the *y*-axis. This indicates that the temperature change only affects ${f}_{\text{fsr}}$ but not fiber dispersion. More details concerning the measurement are found in Note 3 in Supplementary Materials. Therefore, once the ambient temperature is known, the ${f}_{\text{fsr}}$ at 1490 nm can be calculated, as well as the ${f}_{\text{fsr}}$ variation over frequency.

Finally, to verify the measurement reproducibility, the ${f}_{\text{fsr}}$ value at 1490 nm is repeatedly measured 150 times. Figure 2g shows the occurrence histogram, with a standard deviation of 112.5 Hz.

### Performance of the VSA

Here we use a dispersion-calibrated, phase-stable fiber cavity for relative-frequency calibration. We note that frequency comb spectrometers^15,44,45^ with a precisely equidistant grid of frequency lines can also be used^46,47^. While frequency combs have been a proven technology for spectroscopy^48^ with unparalleled accuracy, they have several limitations in the characterization of passive devices. First, in addition to being bulky and expensive, commercial fiber laser combs as spectrometers suffer from limited frequency resolution due to the RF-rate comb line spacing (typically above 100 MHz). Second, the simultaneous injection of more than 10^5^ comb lines can saturate or blind the photodetector, yielding a severely deteriorated signal-to-noise ratio (SNR) and dynamic range.

Different from frequency combs, CW lasers featuring high photon flux and ever-increasing frequency tunability and agility are particularly advantageous for sensing^49^. In our method, after frequency-calibration by the fiber cavity, the chirping CW laser behaves as a frequency comb with a “moving” narrow-band-pass filter, where the filter selects only one comb line each time and rejects other lines. Therefore the nearly constant laser power during chirping provides a flat power envelope over the entire spectral bandwidth. Consequently our method avoids photodetector saturation and device damage. It also increases SNR and dynamic range.

To improve frequency resolution, the extrapolation of instantaneous laser frequency between two neighboring fiber cavity’s resonances is performed, which relies on the frequency linearity of the chirping laser. Such linearity is experimentally characterized in a parallel work^50^ of ours, where the chirping ECDL (Santec TSL) is referenced to a commercial optical frequency comb. The result from ref. ^50^ evidences that the chirping linearity is better for a higher chirp rate. We experimentally test different laser chirp rates, including 20, 50, 100, and 200 nm/s. When combined with a 55-MHz-FSR fiber cavity, the accuracy of linear interpolation within the fiber cavity’s “dead zone” achieves a precision better than 200 kHz for a chirp rate exceeding 50 nm/s, which surpasses the laser linewidth. Meanwhile, if the chirp rate is too high, such that the time that the chirping laser sweeps across the DUT’s resonance is comparable to the resonance lifetime, the measured transmission curve is distorted due to the cavity’s ringdown effect. Taking all these issues into consideration, the chirp rate of 50 nm/s is the most appropriate value in our experiment. More details are elaborated in Note 4 in Supplementary Materials.

The ultimate frequency resolution of each individual time-domain trace is determined by the chirp range divided by the oscilloscope’s memory depth ($2\times {10}^{8}$). For the ECDL of the widest spectral range from 1480 to 1640 nm (19.8 THz), we estimate that the ultimate frequency sample resolution of our VSA is around 99 kHz, i.e. the frequency interval between two recorded neighboring data points. The actual resolution can be compromised by the chirping laser’s linewidth. We measure the dynamic laser linewidth using a self-delayed heterodyne setup. Experimental details are elaborated in Note 5 in Supplementary Materials. Within a 100 μs time scale, the ECDL’s dynamic linewidth at 50 nm/s chirp rate is averaged as 471 kHz. This linewidth is due to multiple reasons, including laser intrinsic linewidth, laser chirp nonlinearity, and the fiber delay-line’s instability in the heterodyne setup. The measured laser dynamic linewidth of 471 kHz sets the lower bound of our VSA’s frequency resolution.

Finally, we compare our method for relative frequency calibration using a fiber cavity with the commonly used method based on an unbalanced Mach–Zehnder interferometer (UMZI)^37,47^. The transmission of a UMZI versus frequency is a sinusoidal curve, which theoretically could provide frequency calibration at any instant. However, it is challenging to measure the local FSR of a UMZI with a precision better than 1 kHz. Thus, to reduce the accumulative error, an optical frequency comb is required^47^. On the other hand, practically, when the laser chirping nonlinearity heavily distorts the sinusoidal curve, extraction of the actual phase at arbitrary instants becomes infeasible. In comparison, it is easier and more reliable to obtain the center dip of a narrow resonance of ~1 MHz linewidth for a fiber cavity. These two points mark the advantages of the fiber cavity over the UMZI for relative frequency calibration.

### Characterization of passive integrated devices

Next we demonstrate several applications using our VSA. We first use our VSA as an OVNA to characterize passive devices. We select two types of optical devices: an integrated optical microresonator and a meter-long spiral waveguide. Both devices, fabricated on silicon nitride (Si_3_N_4_, see Materials and Methods)^51^, have been extensively used in integrated nonlinear photonics^14,16^. For example, optical microresonators of high quality (*Q*) factors are central building blocks for microresonator-soliton-based optical frequency combs (“soliton microcomb”)^13–15^, ultralow-threshold optical parametric oscillators^16,18,19^, and quantum frequency translators^20,21^. Ultralow-loss, dispersion-flattened waveguides are cornerstones for multi-octave supercontinua^22–25^ and continuous-traveling-wave optical parametric amplifiers^26–28^. All these applications require precisely characterized properties of integrated devices, such as loss, phase, and dispersion over a bandwidth spanning more than 100 nm.

**Fig. 3:**
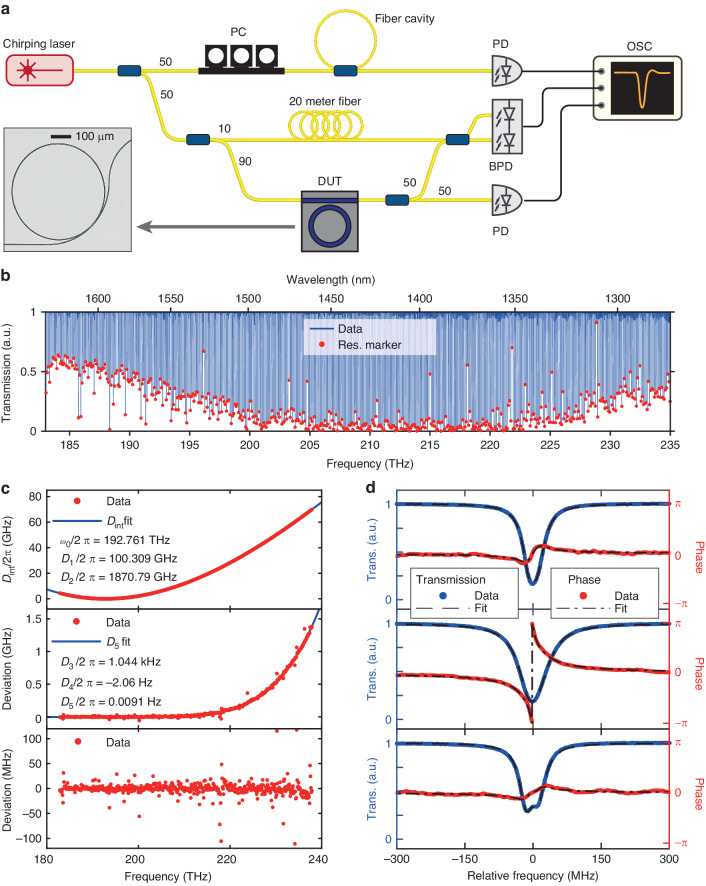
Characterization of passive Si_3_N_4_ microresonators. **a** Experimental setup to measure the transmission (loss), dispersion and phase response of Si_3_N_4_ microresonators. Inset is an optical microscope image showing a Si_3_N_4_ microresonator coupled with a bus waveguide. BPD, balanced photodetector. **b** A typical broadband transmission spectrum of an integrated Si_3_N_4_ microresonator. **c** Measured integrated microresonator dispersion profile and fit up to the fifth order. **d** Measured transmission and phase profiles of three resonances that are under-coupled (top), over-coupled (middle), or feature mode split (bottom, also under-coupled)

Figure 3a shows an optical microscope image of a Si_3_N_4_ microresonator, which is a microring side-coupled by a bus waveguide for optical input and output^51^. The resonance frequency $\omega /2{\rm{\pi }}$ and linewidth $\kappa /2{\rm{\pi }}$ are often the most critical parameters that reveal the waveguide’s dispersion, loss, and phase response. Experimentally, we measure these two parameters of each fundamental-mode resonance, ranging from 1260 nm (237.9 THz) to 1640 nm (182.8 THz) wavelength. First, we extract the integrated dispersion of the Si_3_N_4_ microresonator, which is defined as

1 $${D}_{\mathrm{int}}\left(\mu \right)={\omega }_{{\rm{\mu }}}-{\omega }_{0}-{D}_{1}\mu =\mathop{\sum }\limits_{n=2}^{\cdots }\frac{{D}_{n}{\mu }^{n}}{n!}$$

where ${\omega }_{\mu }/2{\rm{\pi }}$ is the *μ*th resonance frequency relative to the reference resonance frequency ${\omega }_{0}/2{\rm{\pi }}$, ${D}_{1}/2{\rm{\pi }}$ is microresonator’s FSR, ${D}_{2}/2{\rm{\pi }}$ describes group velocity dispersion (GVD), and *D*_*n*__≥3_ are higher-order dispersion terms. Experimentally, as shown in Fig. 3a, half of the laser power is split for frequency calibration with the fiber cavity, and the other half is then split into two branches. In the lower branch, half of the light transmitted through the DUT is directly detected by a photodetector and recorded with an oscilloscope. The microresonator transmission over 55.1 THz optical bandwidth (from 1260 to 1640 nm) is shown in Fig. 3b. Exact resonant frequencies ${\omega }_{\mu }$ are determined with dips searching on the transmission data-trace.

Figure 3c top plots the measured ${D}_{\mathrm{int}}$ profile, with each parameter extracted from the fit using Eq. (1). We note that, due to our 55.1 THz measurement bandwidth and 471 kHz frequency resolution, our method can measure higher-order dispersion^52^ up to the fifth-order (*D*_5_) term. This is validated in Fig. 3c middle, where *D*_3_ and *D*_4_ terms are subtracted from ${D}_{\mathrm{int}}$, and the residual dispersion is fitted with ${D}_{5}{\mu }^{5}/120$. Figure 3c bottom shows that, after further subtraction of the *D*_5_ term, no prominent residual dispersion is observed. Some data points deviate from the fit due to avoided mode crossings in the microresonator^53^.

Next, we fit each resonance to obtain their linewidths. For each resonance fit^54^, the intrinsic loss ${\kappa }_{0}/2{\rm{\pi }}$, external coupling strength ${\kappa }_{\text{ex}}/2{\rm{\pi }}$, and the total (loaded) linewidth $\kappa /2{\rm{\pi }}=\left({\kappa }_{0}+{\kappa }_{\text{ex}}\right)/2{\rm{\pi }}$, are extracted. Figure 3d shows three typical resonances with fit curves (blue), including one with visible mode split (bottom). Conventionally, based on a single resonance profile, it is impossible to judge whether the resonance is over-coupled (${\kappa }_{\text{ex}} > {\kappa }_{0}$) or under-coupled (${\kappa }_{\text{ex}} < {\kappa }_{0}$)^55^. The coupling condition can only be revealed by phase (vector) measurement. As shown in Fig. 3a, since the light exiting the DUT carries an additional phase shift *φ*, we interfere it with the light from the reference upper branch to extract *φ*. To improve the signal-to-noise ratio, a delay Δ*τ* caused by a 20-meter-long fiber is brought into the reference branch. The frequency difference $\Delta f=\gamma \Delta \tau$ between the two branches corresponds to the frequency of the beatnote signal readout with the balanced photodetector, where *γ* is the laser chirp rate. The extra phase shift *φ* also applies to the beat signal, which can be extracted with Hilbert transformation^56^ (see Note 6 in Supplementary Materials). The measured and fitted phases are shown in Fig. 3d red curves. The continuous phase transition across the resonance in Fig. 3d top and bottom represents under-coupling, while the phase jump by 2*π* in Fig. 3d middle represents over-coupling. From top to bottom, the fitted loss values (*κ*_0_/2*π*, *κ*_ex_/2*π*) for each resonance are (23.8, 14.0), (19.9, 42.4), and (24.7, 12.8) MHz. The complex coupling coefficient^57^ in the bottom is *κ*_c_/2*π* = 29.1 + 2.25*i* MHz.

**Fig. 4:**
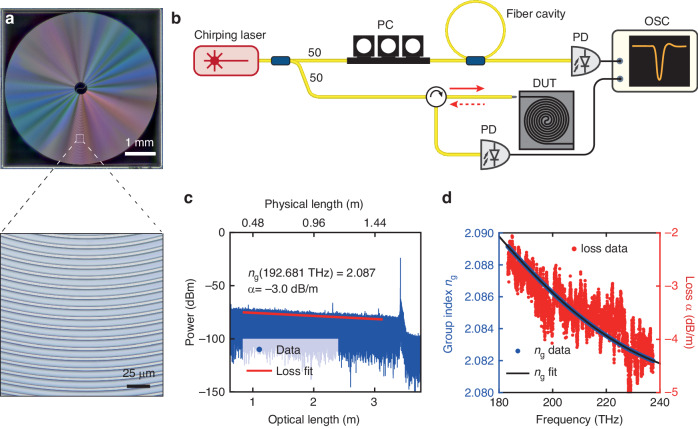
Characterization of meter-long Si_3_N_4_ spiral waveguides. **a** Optical microscope image showing a 1.6394-meter-long spiral waveguide contained in a photonic chip of 5 × 5 mm^2^ size. The zoom-in shows the densely coiled waveguide. **b** Experimental setup of OFDR. The red solid and dashed arrows represent the incident light to and the reflected light from the DUT, respectively. **c** Measured OFDR data of the spiral waveguide. The major peak at 1.6394 m physical length (3.4214 m optical length) is attributed to the light reflection at the rear chip facet, where the waveguide terminates. This length difference indicates a group index of ${n}_{{\rm {g}}}=2.087$ at 192.681 THz. The loss rate $\alpha =-3.0$ dB/m (physical length) is calculated with a linear fit of power decrease over distance (red line). **d** Measured group index *n*_g_ (blue dots), the fit (black line), and loss *α* (red dots) of the waveguide over the 55.1 THz spectral range

In addition to microresonators as well as other resonant structures, our method can also characterize single-pass waveguides. Here we apply our method to measure the linear loss and frequency-dependent group refractive index of integrated waveguides. Figure 4a shows an optical microscope image of a Si_3_N_4_ photonic chip containing a spiral waveguide of ${L}_{0}=1.6394\,{\rm {m}}$ physical length. We use our VSA as optical frequency-domain reflectometry (OFDR)^58^ to characterize the waveguide loss and dispersion. The setup is shown in Fig. 4b. The laser is coupled into the DUT, and the reflected light from the DUT is collected by an AC-coupled photodetector through a circulator. The reflected light at distance *l* within the waveguide of physical length *L*_0_ has an amplitude *A*_*l*_. The strong reflection signal from the waveguide front facet (i.e. $l=0$) has a constant amplitude *A*_0_. Together these two fields *A*_*l*_ and *A*_0_ interfere and create a beat signal of frequency $\Delta f=2\gamma {n}_{{\rm {g}}}l/c$, where *c* is the speed of light in air, *γ* is the laser chirp rate, and *n*_g_ is the group index. Thus, the photodetected beat signal is ${V}_{l}\left(t\right)={A}_{0}{A}_{l}\cos \left(2{\rm{\pi }}\Delta {ft}\right)$. The instantaneous laser chirp rate *γ* is extracted by the relative frequency calibration using the fiber cavity. With FFT, the relative reflection power from different locations in the waveguide is calculated. Figure 4c plots the OFDR signal from the spiral waveguide. The prominent peak located at 1.6394 m physical length (3.4214 m optical length) is attributed to the light reflection at the rear facet of the chip, where the waveguide terminates. The difference in the physical and optical lengths indicates a group index of ${n}_{{\rm {g}}}=2.087$ at 192.681 THz.

In the presence of waveguide dispersion, the optical path length *L*_op_ varies due to the frequency-dependent *n*_g_. This dispersion-induced optical path variation leads to deteriorated spatial resolution in broadband measurement^59^. By dividing the broadband measurement data into narrow-band segments^60,61^, the optical path length at different optical frequencies can be obtained, and thus the frequency-dependent *n*_g_ over the 55.1 THz spectral range can be extracted. With the extracted *n*_g_, the waveguide dispersion can be de-embedded with a re-sample algorithm^61,62^.

Light traveling in the waveguide experiences attenuation following the Lambert–Beer Law $I\left(L\right)={I}_{0}\cdot \exp \left(\alpha L\right)$. In Fig. 4c, the average linear loss $\alpha =-3.0$ dB m^−1^ (physical length) is extracted by applying a first-order polynomial fit of the power profile (red line) within the 19.8 THz bandwidth and centered at 192.681 THz. Figure 4d shows the frequency-dependent *α* (red dots) and *n*_g_ (blue dots) extracted using segmented OFDR algorithm^60,63^. The *n*_g_ is further fitted at 208.015 THz, and the dispersion parameters are extracted up to the fourth order as *β*_1_ = 6955.0 fs/mm, *β*_2_ = −74.09 fs^2^/mm, *β*_3_ = 199 fs^3^/mm, and ${\beta }_{4}=2.4\times {10}^{2}$ fs^4^/mm. The loss fluctuation with varying frequency is likely due to multi-mode interference in the spiral waveguide^64^.

In OFDR, the resolution $\delta {L}_{\text{op}}$ of optical path length is determined by the laser chirp bandwidth *B* as $\delta {L}_{\text{op}}=c/2B$, with *c* being the speed of light in vacuum. Our VSA can provide a maximum $B=19.8$ THz in a single measurement, which enables $\delta {L}_{\text{op}}=7.6$ μm. As shown in Fig. 4c, such a fine resolution allows unambiguous discrimination of scattering points in the waveguide, which are revealed by small peaks. Thus our VSA is proved as a useful diagnostic tool for integrated waveguides.

### Characterization of a soliton spectrum

Next, we use our VSA as an OSA to characterize broadband laser spectra. While modern OSAs can achieve wide spectral bandwidth, they suffer from a limited frequency resolution ranging from sub-gigahertz to several gigahertz. This issue prohibits OSAs from resolving fine spectral features. For example, individual lines of mode-locked lasers or supercontinua with repetition rates in the RF domain cannot be resolved by OSAs. Soliton microcombs with terahertz-rate repetition rate can be useful for low-noise terahertz generation^65,66^, but their precise comb line spacing can neither be measured by normal photodetectors nor OSAs.

**Fig. 5:**
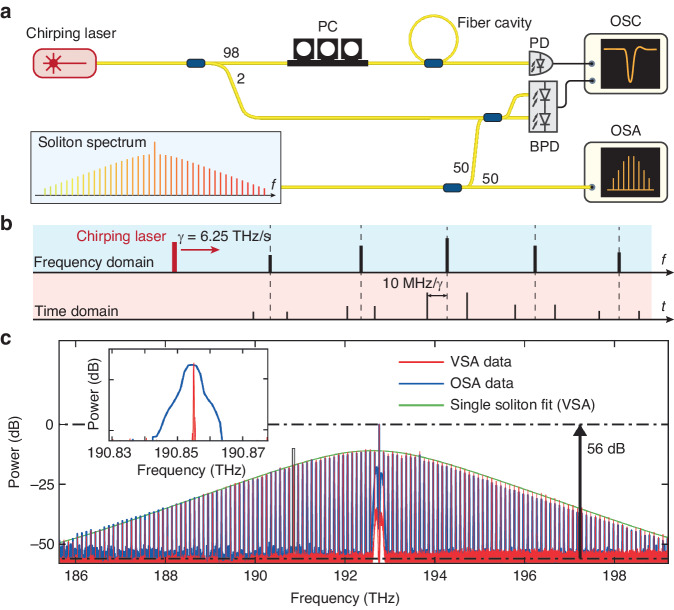
Characterization of a broadband soliton comb spectrum. **a** Experimental setup. OSA, optical spectrum analyzer. **b** Principle of coherent detection of a broadband optical spectrum using a chirping laser. The laser beats progressively with different frequency components of the optical spectrum, which allows frequency detection in the RF domain and continuous information output in the time domain. **c** Single soliton spectra measured by our VSA (red) and a commercial OSA (blue). The spectral envelope of VSA data is fitted with a sech^2^ function (green). Inset: Zoom-in of the comb line resolved by our VSA and the OSA, demonstrating a significant resolution enhancement by the VSA

Here we demonstrate that our VSA can act as an OSA, which features 55.1 THz spectral range and megahertz frequency resolution. As an example, we measure the repetition rate (line spacing) of a soliton microcomb. The experimental setup is shown in Fig. 5a, where the generated soliton interferes with the chirping laser. The interference is then photodetected with a balanced photodiode and recorded with an oscilloscope. The schematic is depicted in Fig. 5b when the laser chirps across the entire soliton spectrum. Every time the laser passes through a comb line, it generates a moving beatnote. Using a digitally implemented finite impulse response (FIR) band-pass filter of 10 MHz center frequency and 3 MHz bandwidth, the beatnote creates a pair of marker signals when the laser frequency is ±10 MHz distant from the comb line. The polarization of the soliton spectrum is measured by varying the laser polarization until the beat signal with maximum intensity is observed. Since the instantaneous laser frequency is precisely calibrated, the comb line spacing is extracted by calculating the frequency distance from two adjacent pairs of marker signals. With the known laser power and measured marker signals’ intensity, the absolute power of each comb line can be calculated. To further measure the relative power of each comb line, we measured the output power of the chirping laser ${P}_{\text{c}}\left(f\right)\propto {\left|{A}_{\text{c}}\left(f\right)\right|}^{2}$ over its entire chirp range. Note that the amplitude of the beatnote signal $\left|{A}_{\text{rf}}\left(f\right)\right|$ is proportional to $\left|{A}_{\text{c}}\left(f\right){A}_{\text{LUT}}\left(f\right)\right|$. Therefore the amplitude of the LUT $\left|{A}_{\text{LUT}}\left(f\right)\right|$ can be calculated.

Figure 5c compares the measured soliton microcomb spectra using our VSA and a commercial OSA. Both spectra are nearly identical, which validates our VSA measurement. The dynamic range of our VSA is found as 56 dB, on par with modern commercial OSAs with the finest resolution (e.g. 45–60 dB at 0.02 nm resolution for Yokogawa OSAs). Figure 5c inset evidences that our VSA indeed provides significantly finer frequency resolution than the OSA’s. The soliton repetition rate measured by the VSA is $\left(100.307\pm 0.002\right)$ GHz.

We emphasize that, here the frequency resolution of our VSA as an OSA is limited by the bandwidth of FIR band-pass filters. In digital data processing, we find that 3 MHz FIR bandwidth yields the optimal resolution bandwidth of 3 MHz. Experimentally, we verify the resolution bandwidth by phase-modulating a low-noise fiber laser (NKT Koheras) to generate a pair of sidebands of 3 MHz difference to the carrier. The carrier and the sidebands are unambiguously resolved using our VSA (see Note 5 in Supplementary Materials). The 3 MHz resolution bandwidth is also consistent with the uncertainty of the measured soliton repetition rate of 100.307 GHz.

### High-resolution LiDAR application

**Fig. 6:**
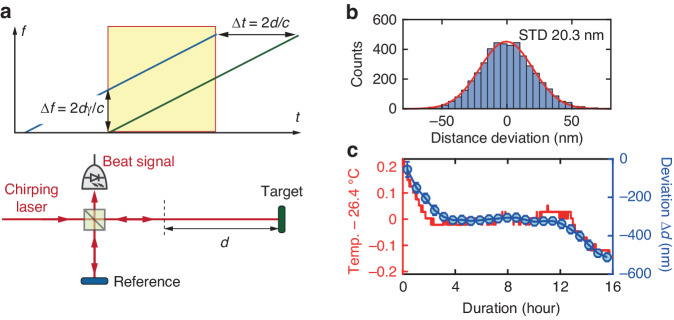
Demonstration of coherent LiDAR. **a** Principle of coherent LiDAR using a linearly chirping laser. With the known chirp rate *γ*, the heterodyne measurement of the beat frequency in the RF domain $\Delta f=2d\gamma /c$ allows calculation of the time delay $\Delta t=2d/c$ and thus the distance *d*. **b** The histogram showing the deviations of 4625 LiDAR measurements from their mean values. The LiDAR precision is revealed by the standard deviation of 20.3 nm. **c** LiDAR measurement of thermal expansion of our optical table using our VSA, in comparison with data from a digital ambient thermometer

Finally, we note that the broadband, chirping, and interferometric nature of our VSA also enables coherent LiDAR. Frequency-modulated continuous-wave (FMCW) LiDAR is a ranging technique based on frequency-modulated interferometry^67^, as depicted in Fig. 6a. The chirping laser is split into two arms, with one arm to the reference and the other to the target with a path difference of *d*. When the reflected signals from both arms recombine at the photodetector, the detected beat frequency is determined as $\Delta f=2d{\rm{\gamma }}/c$, where *c* is the speed of light in air and *γ* is the chirp rate. Thus the measurement of Δ*f* in the RF domain allows distance measurement of *d*. The ranging resolution δ*d*, i.e. the minimum distance that the LiDAR can distinguish two nearby objects, is limited by the chirp bandwidth *B* as $\delta d=c/2B$. One advantage of our VSA as an FMCW LiDAR is that our laser can provide maximum $B=19.8$ THz that enables ${\rm{\delta }}d=7.6$ μm.

In our LiDAR experiment, we set the linear chirp rate of $\gamma =6.25$ THz/s and duration of $T=0.4$ s. The experimental setup and data analysis procedure are found in Note 7 in Supplementary Materials. As a demonstration, we monitor the thermal expansion of our optical table due to ambient temperature drift, as shown in Fig. 6c. The distance difference between the target mirror and the reference mirror on the table is $d=137.63128$ mm. The measured distance change Δ*d* within the 500 nm range agrees with the temperature decrease that causes the contraction of the optical table. After subtracting the global trend, Fig. 6b shows the histogram of the deviations of 4625 measurements from their mean values. Our LiDAR precision is revealed by the standard deviation of 20.3 nm. Such precision is provided by the careful relative-frequency calibration and long-term stability of our VSA.

## Discussion

In summary, we have demonstrated a dual-mode VSA featuring 55.1 THz spectral bandwidth, 471 kHz frequency resolution, and 56 dB dynamic range. The VSA can operate either as an OVNA to characterize the LTF and dispersion property of passive devices, or as an OSA to characterize broadband frequency comb spectra. A comparison of our VSA with other state-of-the-art OSAs and OVNAs is shown in Note 5 in Supplementary Material. Our VSA can also perform LiDAR with distance resolution of 7.6 μm and precision of 20.3 nm. Meanwhile, our VSA is fiber-based. It neither requires high-speed modulators and photodetectors, nor any active feedback control. Therefore the system is compact, robust, and transportable for field-deployable applications.

There are several aspects to further improve the performance of our VSA. First, the frequency accuracy can be improved by adding a highly stable reference laser in the system. When the ECDL scans through the reference laser, the two lasers beat and create a marker in the time-domain trace. The marker marks the point where the chirping ECDL has an instantaneous frequency as the reference laser’s frequency. Second, more ECDLs can be added to the system, allowing further extension of the spectral bandwidth and operation in other wavelength ranges, such as the visible and mid-infrared bands. Meanwhile, even ECDLs with mode hopping can be used in our VSA. The self-calibration and compensation of mode hopping can be realized by adding a calibrated, large-FSR cavity (e.g. a Si_3_N_4_ microresonator of terahertz-rate FSR), in addition to the fine-tooth fiber cavity. By measuring the resonance-to-resonance frequency and referring to the previously calibrated local FSR of the microresonator, the exact mode hopping range and location can be inferred. Adding more calibrated cavities of different FSR values to form a Vernier structure can further enhance precision and accuracy.

Besides the characterization of passive elements and broadband laser sources for integrated photonics, our VSA can also be applied for time-stretched systems^2^, optimized optical coherent tomography (OCT)^68^, linearization of FMCW LiDAR^69^, and resolving fine structures in Doppler-free spectroscopy^70^. Therefore, our VSA presents an innovative approach for device analysis and laser spectroscopy, and can play a crucial role in future photonic systems and applications for sensing, communication, imaging, and quantum information processing.

## Materials and methods

### Device fabrication

The Si_3_N_4_ microresonator and meter-long spiral waveguides are fabricated using a foundry-standard process^46^. It is a subtractive process on 6-inch wafers. First, Si_3_N_4_ and SiO_2_ films are deposited on the thermal wet SiO_2_ substrate via low-pressure chemical vapor deposition (LPCVD). Deep-ultraviolet (DUV) stepper photolithography (248 nm KrF) is used to expose the waveguide pattern. The pattern is subsequently transferred from the photoresist mask to the SiO_2_ hard mask and then to the Si_3_N_4_ layer via dry etching. Then the etched substrate is thermally annealed in a nitrogen atmosphere at 1200 °C to remove hydrogen contents in Si_3_N_4_. Top SiO_2_ cladding is then deposited on the wafer, followed by another high-temperature annealing. Finally, contact photolithography and deep etching of SiO_2_ and Si are used to divide a wafer into hundreds of chips, followed by dicing.

### Supplementary information

Supplementary Materials

## Data Availability

The code and data that support the findings of this study are openly available in Zenodo (10.5281/zenodo.10803802).

## References

[1] Yang ZY (2021). Miniaturization of optical spectrometers. Science.

[2] Mahjoubfar A (2017). Time stretch and its applications. Nat. Photonics.

[3] Li A (2022). Advances in cost-effective integrated spectrometers. Light Sci. Appl..

[4] Ryckeboer E (2013). Silicon-on-insulator spectrometers with integrated GaInAsSb photodiodes for wide-band spectroscopy from 1510 to 2300 nm. Opt. Express.

[5] Pohl D (2020). An integrated broadband spectrometer on thin-film lithium niobate. Nat. Photonics.

[6] Yang ZY (2019). Single-nanowire spectrometers. Science.

[7] Yoon HH (2022). Miniaturized spectrometers with a tunable van der Waals junction. Science.

[8] Zhang ZY (2022). Integrated scanning spectrometer with a tunable micro-ring resonator and an arrayed waveguide grating. Photonics Res..

[9] Ni YB (2022). Computational spectropolarimetry with a tunable liquid crystal metasurface. eLight.

[10] Li YH (2023). A platform for integrated spectrometers based on solution-processable semiconductors. Light Sci. Appl..

[11] Cen QQ (2023). Microtaper leaky-mode spectrometer with picometer resolution. eLight.

[12] Toulouse A (2021). 3D-printed miniature spectrometer for the visible range with a 100 × 100 μm^2^ footprint. Light: Adv. Manuf..

[13] Kippenberg TJ (2018). Dissipative Kerr solitons in optical microresonators. Science.

[14] Gaeta AL, Lipson M, Kippenberg TJ (2019). Photonic-chip-based frequency combs. Nat. Photonics.

[15] Diddams SA, Vahala K, Udem T (2020). Optical frequency combs: coherently uniting the electromagnetic spectrum. Science.

[16] Moss DJ (2013). New CMOS-compatible platforms based on silicon nitride and Hydex for nonlinear optics. Nat. Photonics.

[17] Shu HW (2022). Microcomb-driven silicon photonic systems. Nature.

[18] Lu XY (2020). On-chip optical parametric oscillation into the visible: generating red, orange, yellow, and green from a near-infrared pump. Optica.

[19] Perez EF (2023). High-performance Kerr microresonator optical parametric oscillator on a silicon chip. Nat. Commun..

[20] Li Q, Davanço M, Srinivasan K (2016). Efficient and low-noise single-photon-level frequency conversion interfaces using silicon nanophotonics. Nat. Photonics.

[21] Lu XY (2019). Chip-integrated visible–telecom entangled photon pair source for quantum communication. Nat. Phys..

[22] Johnson AR (2015). Octave-spanning coherent supercontinuum generation in a silicon nitride waveguide. Opt. Lett..

[23] Porcel MAG (2017). Two-octave spanning supercontinuum generation in stoichiometric silicon nitride waveguides pumped at telecom wavelengths. Opt. Express.

[24] Carlson DR (2017). Self-referenced frequency combs using high-efficiency silicon-nitride waveguides. Opt. Lett..

[25] Guo HR (2018). Mid-infrared frequency comb via coherent dispersive wave generation in silicon nitride nanophotonic waveguides. Nat. Photonics.

[26] Pu MH (2018). Ultra-efficient and broadband nonlinear AlGaAs-on-insulator chip for low-power optical signal processing. Laser Photonics Rev..

[27] Ye ZC (2021). Overcoming the quantum limit of optical amplification in monolithic waveguides. Sci. Adv..

[28] Riemensberger J (2022). A photonic integrated continuous-travelling-wave parametric amplifier. Nature.

[29] Qu Y (2023). Integrated optical parametric amplifiers in silicon nitride waveguides incorporated with 2D graphene oxide films. Light: Adv. Manuf..

[30] Zhou ZC (2023). Prospects and applications of on-chip lasers. eLight.

[31] Xu HN (2023). Breaking the resolution-bandwidth limit of chip-scale spectrometry by harnessing a dispersion-engineered photonic molecule. Light Sci. Appl..

[32] Foster MA, Moll KD, Gaeta AL (2004). Optimal waveguide dimensions for nonlinear interactions. Opt. Express.

[33] Goda K, Jalali B (2013). Dispersive Fourier transformation for fast continuous single-shot measurements. Nat. Photonics.

[34] Sandel D (1998). Optical network analysis and longitudinal structure characterization of fiber Bragg grating. J. Lightwave Technol..

[35] Gifford DK (2005). Optical vector network analyzer for single-scan measurements of loss, group delay, and polarization mode dispersion. Appl. Opt..

[36] Jin C (2013). High-resolution optical spectrum characterization using optical channel estimation and spectrum stitching technique. Opt. Lett..

[37] Li J (2012). Sideband spectroscopy and dispersion measurement in microcavities. Opt. Express.

[38] Yi XW (2012). Characterization of passive optical components by DSP-based optical channel estimation. IEEE Photonics Technol. Lett..

[39] Tang ZZ, Pan SL, Yao JP (2012). A high resolution optical vector network analyzer based on a wideband and wavelength-tunable optical single-sideband modulator. Opt. Express.

[40] Pan SL, Xue M (2017). Ultrahigh-resolution optical vector analysis based on optical single-sideband modulation. J. Lightwave Technol..

[41] Qing T (2019). Optical vector analysis with attometer resolution, 90-db dynamic range and THz bandwidth. Nat. Commun..

[42] He ZQ (2023). Simple and accurate dispersion measurement of GaN microresonators with a fiber ring. Opt. Lett..

[43] Zhang XB (2021). Dispersion engineering and measurement in crystalline microresonators using a fiber ring etalon. Photonics Res..

[44] Coddington I, Newbury N, Swann W (2016). Dual-comb spectroscopy. Optica.

[45] Yang QF (2019). Vernier spectrometer using counterpropagating soliton microcombs. Science.

[46] Del’Haye P (2009). Frequency comb assisted diode laser spectroscopy for measurement of microcavity dispersion. Nat. Photonics.

[47] Twayana K (2021). Frequency-comb-calibrated swept-wavelength interferometry. Opt. Express.

[48] Picqué N, Hänsch TW (2019). Frequency comb spectroscopy. Nat. Photonics.

[49] Giorgetta FR (2010). Fast high-resolution spectroscopy of dynamic continuous-wave laser sources. Nat. Photonics.

[50] Shi, B. Q. et al. Frequency-comb-linearized, widely tunable lasers for coherent ranging. *Photonics Res.* **12**, 663–681 (2024).

[51] Ye ZC (2023). Foundry manufacturing of tight-confinement, dispersion-engineered, ultralow-losssilicon nitride photonic integrated circuits. Photonics Res..

[52] Yang KY (2016). Broadband dispersion-engineered microresonator on a chip. Nat. Photonics.

[53] Herr T (2014). Mode spectrum and temporal soliton formation in optical microresonators. Phys. Rev. Lett..

[54] Li Q (2013). Unified approach to mode splitting and scattering loss in high-*Q* whispering-gallery-mode microresonators. Phys. Rev. A.

[55] Cai M, Painter O, Vahala KJ (2000). Observation of critical coupling in a fiber taper to a silica-microsphere whispering-gallery mode system. Phys. Rev. Lett..

[56] Marple L (1999). Computing the discrete-time “analytic” signal via FFT. IEEE Trans. Signal Process..

[57] Pfeiffer MHP (2018). Ultra-smooth silicon nitride waveguides based on the damascene reflow process: fabrication and loss origins. Optica.

[58] Soller BJ (2005). High resolution optical frequency domain reflectometry for characterization of components and assemblies. Opt. Express.

[59] Glombitza U, Brinkmeyer E (1993). Coherent frequency-domain reflectometry for characterization of single-mode integrated-optical waveguides. J. Lightwave Technol..

[60] Bauters JF (2011). Planar waveguides with less than 0.1 dB/m propagation loss fabricated with wafer bonding. Opt. Express.

[61] Zhao D (2017). High resolution optical frequency domain reflectometry for analyzing intra-chip reflections. IEEE Photonics Technol. Lett..

[62] Kohlhaas A, Fromchen C, Brinkmeyer E (1991). High-resolution OCDR for testing integrated-optical waveguides: dispersion-corrupted experimental data corrected by a numerical algorithm. J. Lightwave Technol..

[63] Zhao QC (2020). Low-loss low thermo-optic coefficient Ta_2_O_5_ on crystal quartz planar optical waveguides. APL Photonics.

[64] Ji XR (2022). Compact, spatial-mode-interaction-free, ultralow-loss, nonlinear photonic integrated circuits. Commun. Phys..

[65] Tetsumoto T (2021). Optically referenced 300 GHz millimetre-wave oscillator. Nat. Photonics.

[66] Wang BC (2021). Towards high-power, high-coherence, integrated photonic mmWave platform with microcavity solitons. Light Sci. Appl..

[67] Amann MC (2001). Laser ranging: a critical review of unusual techniques for distance measurement. Opt. Eng..

[68] Adler DC (2007). Three-dimensional endomicroscopy using optical coherence tomography. Nat. Photonics.

[69] Roos PA (2009). Ultrabroadband optical chirp linearization for precision metrology applications. Opt. Lett..

[70] Meek SA (2018). Doppler-free Fourier transform spectroscopy. Opt. Lett..

