# Supplementary material for: A wideband, high-resolution vector spectrum analyzer for integrated photonics
Source: Light Sci Appl. 2024 Apr 8;13:83. doi: 10.1038/s41377-024-01435-z (PMC10999422; doi:10.1038/s41377-024-01435-z)
Supplement: Supplementary file 1 — Supplementary Materials [file 41377_2024_1435_MOESM1_ESM.pdf]

Supplementary Materials for: A wideband, high-resolution vector spectrum analyzer for integrated photonics

Yi-Han Luo,[1,2] Baoqi Shi,[1,3] Wei Sun,[1] Ruiyang Chen,[1,2] Sanli Huang,[1,4] Zhongkai Wang,[1] Jinbao Long,[1] Chen Shen,[1] Zhichao Ye,[5] Hairun Guo,[6] and Junqiu Liu[1,4]

[1] *International Quantum Academy, Shenzhen 518048, China*
[2] *Shenzhen Institute for Quantum Science and Engineering, Southern University of Science and Technology, Shenzhen 518055, China*
[3] *Department of Optics and Optical Engineering, University of Science and Technology of China, Hefei, Anhui 230026, China*
[4] *Hefei National Laboratory, University of Science and Technology of China, Hefei 230088, China*
[5] *Qaleido Photonics, Hangzhou 310000, China*
[6] *Key Laboratory of Specialty Fiber Optics and Optical Access Networks, Shanghai University, Shanghai 200444, China*

Supplementary Note 1. Absolute frequency calibration and cascading multiple ECDLs for spectral extension

In our experiment, three ECDLs are cascaded. The respective spectral ranges of the three ECDLs are shown in Fig. S1a, where each two adjacent ECDLs share a common spectral range. In our method, these shared ranges are exploited not only for cascading lasers but also for absolute frequency calibration.

The Santec ECDL has a monitor panel where the value of the current laser frequency is displayed. To set the laser frequency, we send a command to the ECDL via an external computer. Then the laser is set to this frequency, and the frequency value is displayed on the laser's monitor panel.

First, we illustrate the absolute frequency calibration of Laser #1. Initially, the frequency of Laser #2 is set to the Marker #1's frequency at 220.92 THz (1357.0 nm), as shown in the Fig. S1a. Then Laser #1 starts to chirp. When Laser #1's frequency scans across Laser #2's frequency at Marker #1, the beat signal between Lasers #1 and #2 can be detected by the photodetector and recorded by the oscilloscope. In our experiment, the beat signal is filtered by a FIR bandpass filter of 10 MHz center frequency. In this way, two marker signals are created in the Laser #1's time trace due to the beat signal, when Laser #1's frequency is ± 10 MHz detuned to Laser #2's frequency. Therefore the time when Laser #1 chirps across Marker #1's frequency (that is known) can be precisely extracted, which acts as an absolute frequency reference for Laser #1's time trace. Similarly, the Marker #2 set by Laser #3 is used as the absolute frequency marker of Laser #2; The Marker #3 set by Laser #2 is used as the absolute frequency marker of Laser #3. Using the absolute frequency markers described above, together with the relative frequency calibration using the fiber cavity, the frequency-time mapping of the three chirping ECDLs can be individually constructed. Then the three calibrated time traces are seamlessly stitched to form one continuous trace covering the entire 55.1 THz spectral range.

Next, we experimentally measure the three markers' frequency values with a commercial wavelength meter (High-Finesse WS6-200) with an accuracy of 200 MHz and a resolution of 2 MHz. We first test Laser #2 since Markers #1 and #3 are created by Laser #2. The frequency of Laser #2 is repeatedly switched between 1482.0000 nm (202.28911 THz) and 1357.0000 nm (220.92296 THz). For Marker #2 created by Laser #3, the frequency of Laser #3 is repeatedly switched between 1482.0000 nm (202.28911 THz) and 1640.0000 nm (182.80028 THz). The time interval between two subsequent frequency switches is 60 s. During this time interval, the laser frequency is tracked

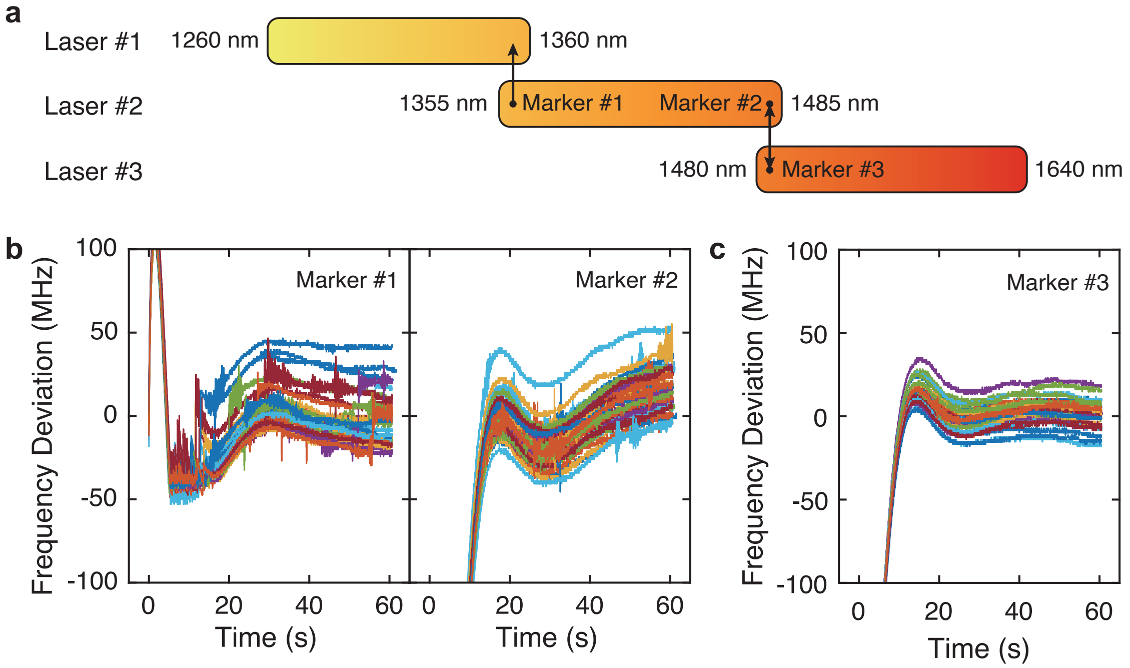

Supplementary Figure S1: laser cascade procedure and absolute frequency calibration. **a**. The spectral ranges of the three ECDLs used in our experiment, and the three selected markers for absolute frequency measurement using a wavelength meter. **b-c**. The frequency drift of the markers is measured by the wavelength meter. For each data trace, the measurement time duration is 60 s. To evidence the marker's frequency deviation, an offset frequency is subtracted. The offset frequency is chosen by averaging all the data in all the traces between 30 to 60 seconds.

TABLE S1: Experimental workflow of the VSA.

Time (s)	Laser Action	Data Process Action
0	Set Laser #2 at 1357.0000 nm	-
60	Laser #1 starts chirping	Oscilloscope starts recording data from PD
63	Set Laser# 3 at 1482.0000 nm	Oscilloscope starts transmitting data to PC
88	-	Frequency calibration of Laser #1 data trace
123	Laser #2 starts chirping	Oscilloscope starts recording data from PD
126	Set laser #2 at 1482.0 nm	Oscilloscope starts transmitting data to PC
151	-	Frequency calibration of Laser #2 data trace
186	Laser #3 starts chirping	Oscilloscope starts recording data from PD
189	Finish	Oscilloscope starts transmitting data to PC
214	-	Frequency calibration of Laser #3 data trace
220	-	Finish

and recorded with the wavelength meter. The key question to answer is, whether the laser frequencies measured by the wavelength meter are precisely equal to the frequency value we set to the lasers.

The measurement results for Lasers #2 and #3 are shown in Fig. S1(b, c). For each marker, a total of 60 traces are measured, and each trace is continuously measured for 60 s. In each trace, the data from 30 s to 60 s are used to calculate the mean frequency. The mean frequency values of Marker #1, #2 and #3, measured by the wavelength meter, are 220.92340(2) THz, 202.28896(2) THz and 202.28901(1) THz, respectively. The differences between the measured frequency values from the set values are 0.44 GHz, 0.15 GHz, and 0.10 GHz. Figure S1(b, c) shows that, for each marker, the deviations of measured frequency values to their mean value are within the accuracy of the wavelength meter (200 MHz). Therefore, in the experiment, we use the measured frequency values of 220.92340 THz, 202.28896 THz, and 202.28901 THz as the markers' frequency values.

The workflow of our experiment procedure is detailed in Table S1. Before the selected ECDL starts chirping, its reference laser is set to the specified frequency and waits 60 s for stabilization. The actual emission frequency of the reference laser is measured by the wavelength meter. After the 60 s, the laser starts chirping. The photodetector probes the laser chirping, and the data is recorded by the oscilloscope. For the other two ECDLs, the operation procedure is similar. Meanwhile, during the 60 s waiting time for laser frequency stabilization, the recorded data by the oscilloscope can be processed by the computer. Finally, the three individually measured and calibrated data traces of each ECDL are stitched, forming the full 55.1 THz spectral bandwidth measurement.

Supplementary Note 2. Details of fiber cavity calibration

In this section, we elaborate the fiber cavity's FSR calibration process.

The CW laser emitted from the ECDL can be written as $A(t) = Ae^{-i2\pi ft}$, where A is the amplitude, f is the frequency. The phase of the CW laser is sinusoidally modulated with a frequency of ν and an amplitude of ϵ. After the phase modulation, the light field obtains an additional time-dependent phase, then the optical field can be expressed as $A(t) = Ae^{-i(2\pi ft+\epsilon\sin 2\pi\nu t)}$. Using the Jacobi–Anger expansion, the optical field can be further written as

$$A(t) = A\sum_m J_m(\epsilon)e^{-i(2\pi f+m2\pi\nu)t}, \tag{S1}$$

where $J_m(z)$ is the m-th Bessel function of the first kind. From the Eq. S1, one can see that, via the phase modulation, a set of CW components with frequencies of $\{f + m\nu \mid m = 0, \pm1, \pm2, \cdots\}$ are generated from the input light.

The modulated CW laser is then sent to the fiber cavity. Practically, the fiber cavity's FSR is much larger than the cavity's resonance linewidth, and the modulation frequency $\nu \approx f_{\mathrm{FSR}}$. The laser frequency f is set as $f \approx f_0$ where f_0 is the fiber cavity's resonant frequency f_0. We limit the frequency difference $|f - f_0|$ on the same order as the corresponding resonance's linewidth. We further assume the response functions of each resonance near f_0 are the same.

Based on the above conditions, the optical field exiting the fiber cavity is written as

$$\begin{aligned} A(t) &= A\sum_m J_m(\epsilon)\mathcal{T}(f + m\nu - f_m)e^{-i(2\pi f+m2\pi\nu)t}, \\ &= A\sum_m J_m(\epsilon)\mathcal{T}(f - f_0 + m\Delta)e^{-i(2\pi f+m2\pi\nu)t}. \end{aligned} \tag{S2}$$

where $\mathcal{T}(\Delta f)$ depicts the lineshape of the resonance, $f_m = f_0 + m f_{\rm fsr}$, and $\Delta = \nu - f_{\rm fsr}$ is the difference between the modulation frequency and the fiber cavity's FSR at f_0. When $\nu = f_{\rm fsr}$, we have $\Delta = 0$, thus

$$\begin{aligned} A(t) &= \mathcal{T}(f - f_0) A \sum_m J_m(\epsilon) e^{-i(2\pi f + m 2\pi\nu)t} \\ &= \mathcal{T}(f - f_0) A e^{-i(2\pi f t + \epsilon \sin 2\pi\nu t)}. \end{aligned} \tag{S3}$$

Therefore, the optical intensity after the fiber cavity is calculated as

$$I(t) = |A(t)|^2 = |A\mathcal{T}(f - f_0)|^2, \tag{S4}$$

which is time-independent. If $\nu \neq f_{\rm FSR}$, the response function $\mathcal{T}(f - f_0 + m\Delta)$ in Eq. S2 is m-dependent. Thus the light intensity $I(t) = |A(t)|^2$ becomes time-dependent, leading to the appearance of a set of beatnote signals of frequency $\{m\nu \mid m = 1, 2, \cdots\}$.

Figure S2a and S2b show an example of numerical simulation. Here the fiber cavity's lineshape is Lorentzian as $\mathcal{T}(\Delta f) = 1 - 1/(1 + i2\pi\Delta f/\kappa)$, where $\kappa/2\pi$ is the resonance linewidth. The parameters are selected as $f = 100$ Hz, $\nu = 2$ Hz, $\epsilon = 0.1$ Hz, $\kappa/2\pi = 0.1$ Hz, and $f - f_0 = 0.05$ Hz. The summation of m is taken from -15 to $+15$. The red lines correspond to the condition $\Delta = 0$, while the blue lines correspond to the condition $\Delta = 0.01$ Hz. Figure S2a shows the light intensity at the fiber cavity's output. For the condition $\Delta \neq 0$, the light intensity becomes time-dependent (blue line) as the result of pure phase modulation (red line). The resulted frequency domain is shown in the Fig. S2b, with a set of beatnote signal of frequency $\{m\nu \mid m = 1, 2, \cdots\}$ (blue line).

In our experiment, it is not mandatory to lock the laser frequency to a specific frequency. Instead, we sweep the laser frequency across the fiber cavity's resonance. Considering the cavity dynamics, the light field calculated above

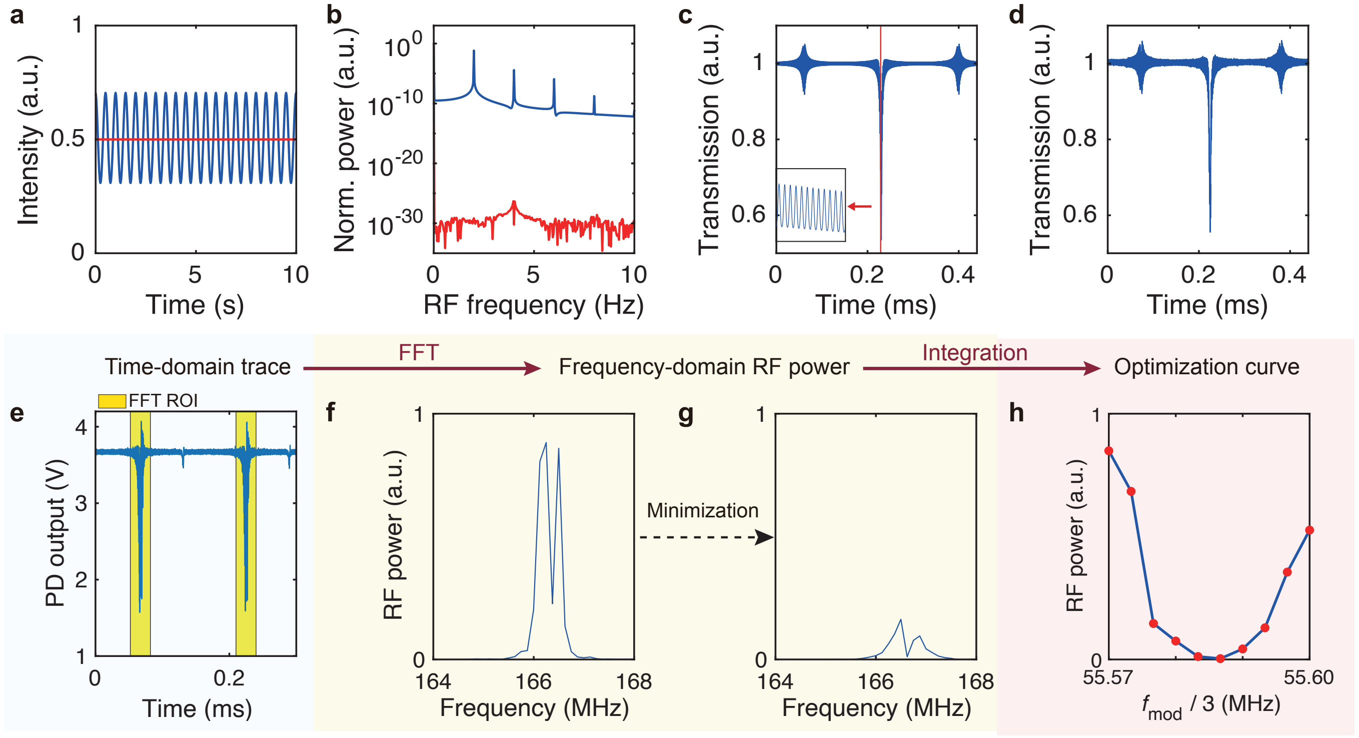

Supplementary Figure S2: Numerical simulation and experimental workflow of the fiber cavity FSR calibration. **a, b**. The optical intensity at the output of the fiber cavity with $f_{\rm mod} = f_{\rm fsr}$ (red) and $f_{\rm mod} \neq f_{\rm fsr}$ (blue), shown both in the time domain (panel a) and in the frequency domain (panel b). When $f_{\rm mod} \neq f_{\rm fsr}$, beatnote signals appear. **c, d**. In the case $f_{\rm mod} = f_{\rm fsr}/2$, during the time that the chirping laser sweeps across the fiber cavity's resonance, the time-domain trace at the output of the fiber cavity is recorded by the oscilloscope. The simulation result (panel c) is consistent with the experimentally measured result (panel d). The inset of panel c shows the zoomed-in beatnote signals in the time domain. **e-h**. The fiber cavity calibration workflow. **e**. The time-domain trace recorded by the oscilloscope. The FFT region of interest (ROI) is selected as neighborhood of each resonance. **f, g**. The FFT result of the time-doamin trace in one FFT ROI. As the $f_{\rm mod}$ approaches $N \cdot f_{\rm fsr}$, the RF power peak at $f_{\rm mod}$ decreases (panel f to g). **h**. The principal parameter to minimize, which is the integration of the RF power peak at $f_{\rm mod}$, versus $f_{\rm mod}$. The modulation frequency that minimizes the total RF power is equal to $N \cdot f_{\rm fsr}$ (here $N = 3$).

within the frequency domain is insufficient. The actual calibration process ought to be described with Langevin equation

$$\frac{\mathrm{d}a_m}{\mathrm{d}t} = -i2\pi f_m a_m - \frac{\kappa_0 + \kappa_{\mathrm{ex}}}{2} a_m + \sqrt{\kappa_{\mathrm{ex}}} a_{\mathrm{in}}, \tag{S5}$$

where κ_0 and κ_{ex} are the intrinsic loss and external coupling strength of the fiber cavity, respectively. a_m is the amplitude of the CW component adjacent to the cavity's resonance of frequency f_m. In the experiment, the laser sweeps from the resonance's blue-detuned side with an initial frequency of $f_0 + \nu_0$ ($\nu_0 > 0$) to its red-detuned side, at a sweeping rate of v. Together with the phase modulation, the chirping input field can be written as $a_{\mathrm{in}} = Ae^{-i2\pi\left[f_0 t + \nu_0 t + v^2 t/2 + (\epsilon/2\pi)\sin\Omega t\right]}$. For a more efficient numerical simulation, the equation is transformed to the frame rotating with frequency f_0 as

$$\frac{\mathrm{d}a_m}{\mathrm{d}t} = -i2\pi m f_{\mathrm{fsr}} a_m - \frac{\kappa_0 + \kappa_{\mathrm{ex}}}{2} a_m + \sqrt{\kappa_{\mathrm{ex}}} A e^{-i2\pi\left[\nu_0 t + v^2 t/2 + (\epsilon/2\pi)\sin\Omega t\right]}. \tag{S6}$$

Runge-Kutta method is used to numerically solve the differential Eq. S6. Then the intra-cavity field evolution $a(t) = \sum_m a_m(t)$ is obtained. The fiber cavity's output is then calculated with the input-output relationship $a_{\mathrm{out}}(t) = a_{\mathrm{in}}(t) - \sqrt{\kappa_{\mathrm{ex}}} a(t)$.

We experimentally measure the sweeping laser input with photodiode, and compare it with numerical result. Figure S2c and S2d show the numerical result and the experimental result, respectively. The modulation frequency ν is selected as $\nu = f_{\mathrm{fsr}}/2$. In this case, the sidebands of the two adjacent resonances overlap and interfere with each other. The numerical result obtained with the Langevin equation is highly consistent with the experimental result. The near-resonant region in Fig. S2c is zoomed in and shown in the inset. Experimentally, we extract the component of intensity oscillation with the frequency of ν with fast Fourier transformation (FFT). As ν approaches the fiber cavity's FSR, the oscillation amplitude decreases. Experimentally, we repeat the calibration process with modified ν values to minimize the RF power at the frequency ν.

In the experiment, the measurement precision is limited. One cannot distinguish the RF power difference for any $\Delta < \epsilon$, where ϵ depicts the precision to determine the modulation frequency f_{mod} for RF power minimization. Thus the modulation frequency ν is considered as $\nu = f_{\mathrm{FSR}}$, which actually can be $\nu \in [f_{\mathrm{fsr}} - \epsilon, f_{\mathrm{fsr}} + \epsilon]$. To improve the measurement precision, the modulation frequency is set to $\nu \approx N f_{\mathrm{fsr}}$. Equation S2 can be further modified to a general form as

$$\begin{aligned} A(t) &= A \sum_m J_m(\epsilon) \mathcal{T}(f + m\nu - f_{Nm}) e^{-i(2\pi f + m2\pi\nu)t}, \\ &= A \sum_m J_m(\epsilon) \mathcal{T}(f - f_0 + m\Delta) e^{-i(2\pi f + m2\pi\nu)t}. \end{aligned} \tag{S7}$$

where $\Delta = \nu - N f_{\mathrm{fsr}}$. The power of the beatnote signal after the fiber cavity depends only on the actual value of Δ. For the same experiment setup and minimization method, the measurement precision is invariant, i.e., the previous defined ϵ. Thus, the frequency ν/N is considered to be f_{fsr}, which actually actually can be $\nu/N \in [f_{\mathrm{fsr}} - \epsilon/N, f_{\mathrm{fsr}} + \epsilon/N]$. Experimentally, the selection of N depends on the bandwidth of the low-noise photodetector (Newport 1811, 125 MHz bandwidth). To compromise the signal-to-noise ratio and the FSR of the calibrated fiber cavity, we finally select $N = 3$.

Figure S2e-S2h illustrates detailed experimental workflow. The chirping laser is phase-modulated at frequency $f_{\mathrm{mod}} \approx N f_{\mathrm{fsr}}$ and sent to the fiber cavity that is to be calibrated. The light exiting the fiber cavity is photodetected and recorded as a time-domain trace, which is shown in the panel e. With the time-domain trace, the FFT region of interest (ROI) is selected as neighborhood of each resonance. The result of the FFT is shown in the panel f and g. As $|\Delta| = |f_{\mathrm{mod}} - N f_{\mathrm{fsr}}|$ decreases, the FFT peak at f_{mod} decreases. We note that a frequency split exists due to the cavity's ringdown dynamic, which is consistent with the simulation result shown in the Fig. 2d in the main text. The total RF power is extracted with integration and used as the principal parameter for optimization of $\Delta = 0$. We repeat the calibration process but with different modulation frequency values, to obtain the optimization curve as shown in the panel h. The modulation frequency that minimizes the total RF power is $N f_{\mathrm{fsr}}$.

Supplementary Note 3. The fiber cavity's dispersion fit and its temperature stability

In the main text, Fig. 2e shows the calibration of the fiber cavity's FSR f_{fsr} that is frequency-dependent. This determines the accuracy of the relative frequency calibration in our VSA. We measure f_{fsr} from 1260 to 1640 nm with

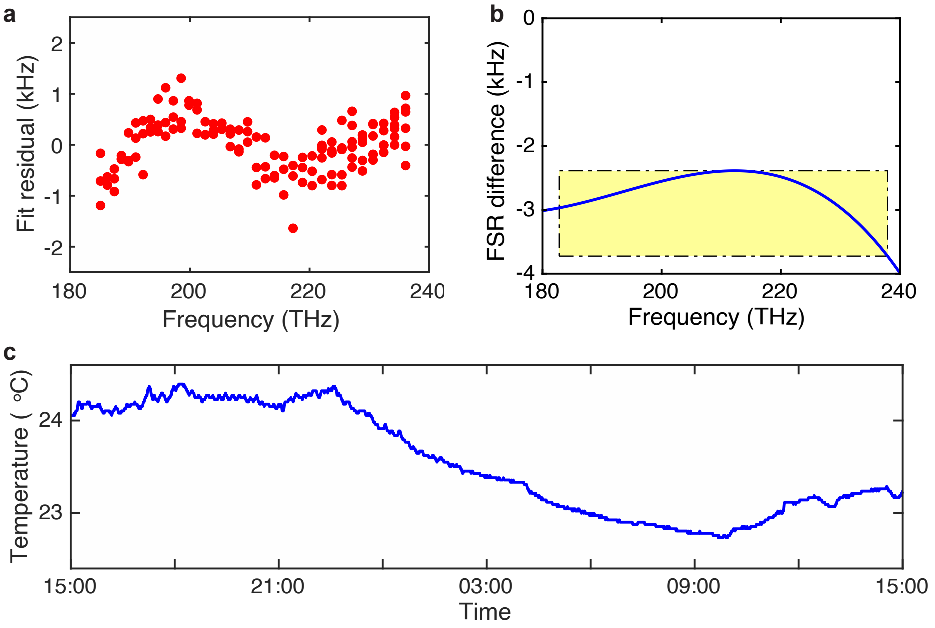

Supplementary Figure S3: Characterization of the fiber cavity's temperature stability. **a**. The fit residual of the fiber cavity's FSR using a quadratic polynomial fit, evidencing the necessity to use a cubic polynomial fit. **b**. The fiber cavity's FSR drifts with $\Delta T = 9.3$ °C temperature change. **c**. Measurement of the ambient temperature in our laboratory for 24 hours.

an interval of 10 nm. The measured data are fitted with a cubic polynomial formula $f_{\mathrm{fsr}}(\nu) = p_1\nu^3 + p_2\nu^2 + p_3\nu + p_4$, where ν is the optical frequency. The fit parameters are: $p_1 = 1.217(11) \times 10^{-7}$, $p_2 = -9.8(7) \times 10^{-5}$, $p_3 = 0.026(2)$, $p_4 = 53.38(11)$. The reason why we use a cubic polynomial fit is the following. If we use a quadratic polynomial fit, the fit residuals (i.e. data deviations from the fit curve) are shown in Fig. S3a. The profile of residuals indicates that, due to our wide spectral measurement, indeed a cubic polynomial fit (to the third order) is necessary.

For the thermal stability test, we place the fiber cavity on a hot plate to investigate the influence of temperature drift. The fiber cavity's temperature is monitored by a thermistor thermometer with a measurement accuracy of 0.5 °C and a resolution of 0.01 °C. The temperature detector is placed on the coiled fiber cavity. Since the ambient temperature in our lab fluctuates within 1 degree over 24 hours, as shown in Fig. S3c, we do not apply any active temperature stabilization on our experimental setup.

As shown in Fig. 2e in the main text, the two measured fiber dispersion curves at different temperatures are nearly identical except with a global relative shift in the y-axis. The FSR shift among the two curves versus frequency is shown in Fig. S3b. The overall FSR shift is around -3 kHz for 9.3°C temperature change, i.e. -0.3 kHz for 1°C change. Therefore, with this measured fiber cavity's FSR drift with temperature, once the ambient temperature is known, we can calculate the f_{fsr} for a given temperature over the 55.1 THz frequency range.

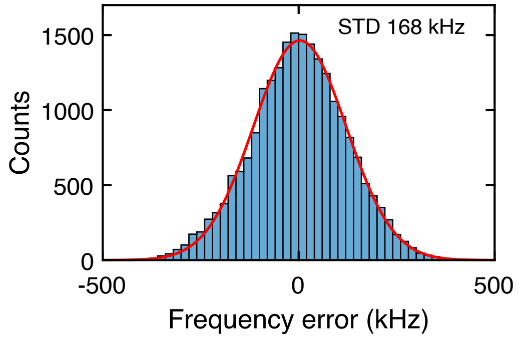

Supplementary Figure S4: Dead zone frequency error. STD, standard deviation.

Supplementary Note 4. Accuracy and precision of the relative frequency calibration

The fiber cavity only indicates the exact relative frequency at the resonances. In the experiment, to calibrate the relative frequency elsewhere, we simply assume the laser chirps linearly within the interval between resonances and utilize a linear interpolation. Thus it is necessary to evaluate the relative calibration accuracy and precision within the "dead zone".

Here we adopt an optical frequency comb (OFC) assisted method[1]. The chirp rate of the Santec laser is set to 50 nm/s. A portion of the chirping laser is split out and interferes with the OFC. The exact laser frequency can then be extracted from the beatnote signal using digital signal processing with a frequency tracking interval of 1 MHz. To evaluate the frequency error introduced by linear interpolation in the "dead zone", we perform linear interpolation every megahertz with the sampled exact frequency at the fiber cavity's resonances. We then compare the OFC measured frequencies with the estimated value obtained by fiber cavity and linear interpolation, the error is statistically shown in Fig. S4 with a histogram. The center of the distribution is 3 kHz, evidencing the interpolation guarantees calibration accuracy and the standard deviation of the distribution reveals the calibration precision is 168 kHz.

Supplementary Note 5. More details on the VSA's performance

In this section, we analyze the performance of the VSA in two aspects: the frequency resolution for passive devices characterizations, and the resolution bandwidth for the spectrum detection.

In an ideal situation where the laser is perfect, the ultimate frequency resolution of the VSA is determined by the memory depth of the oscilloscope and laser chirp range. As stated in the main text, the ultimate frequency resolution is 99 kHz. However, if the laser linewidth is larger than 99 kHz, the data point corresponding to a specific frequency will mix with adjacent ones. The real frequency resolution is determined by the shortest slab of the system. Therefore it is critical to evaluate the laser's dynamic linewidth.

The self-delayed heterodyne setup to measure the laser's dynamic linewidth is shown in Fig S5a. The chirping laser is split into two branches. The upper branch passes through a 20408-meter-long fiber line to introduce a time delay $\Delta\tau$, which is longer than the coherent time of the laser. The upper branch is then combined with the undelayed

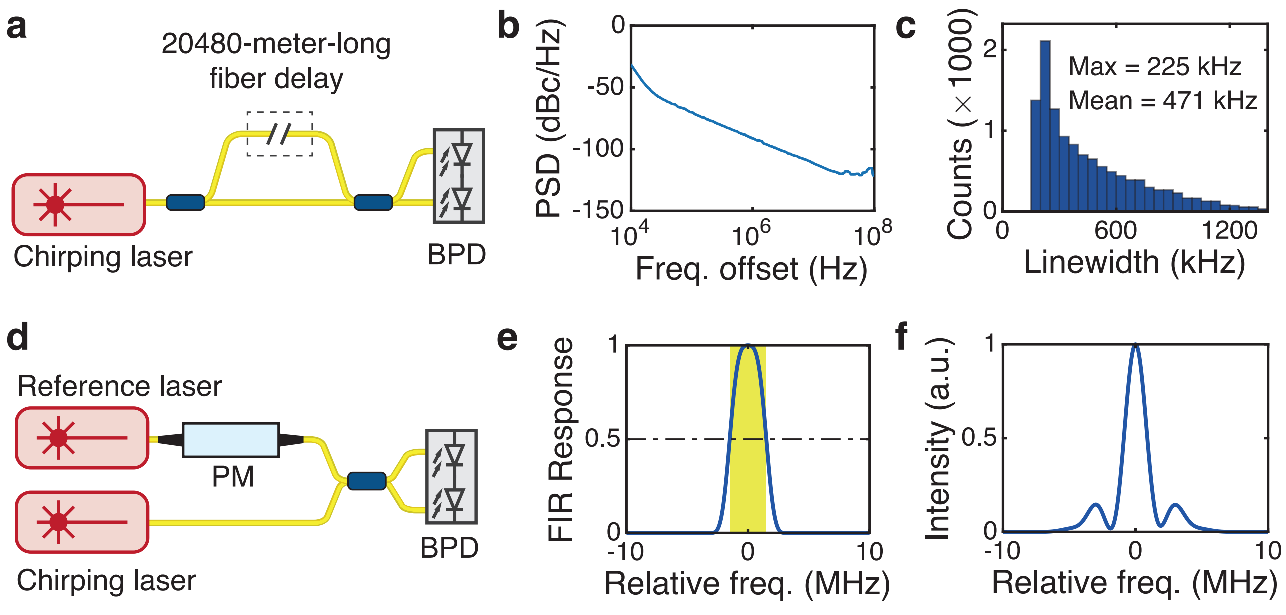

Supplementary Figure S5: Performance characterization of our VSA. a. The self-delayed heterodyne measurement setup. **b**. Measurement of the static single-sideband frequency noise of the chirping laser. **c**. The histogram of the chirping laser dynamic linewidth measured results, where 12207 100-μs segments of beatnote signals are taken and processed with FFT to extract the linewidth. **d**. The setup to verify the resolution bandwidth of spectrum dection of our VSA. **e**. The frequency response of the FIR filter used in the VSA spectral measurement. **f**. The spectrum of phase modulated reference laser measured by our VSA, where two sidebands 3 MHz from the carrier can be unambiguously observed.

TABLE S2: Performance comparison between multiple state-of-the-art OSAs, OVNAs, and our VSA.

Measure Mode		YOKOGAWA AQ6380	II–VI 1500S	EXFO CTP10	APEX AP208xB	OVNA[2]	This work
Loss & Phase	Bandwidth	-	-	52.9 THz	-	1.075 THz	55.1 THz
	Resolution	-	-	125 MHz	-	334 Hz	99 kHz
Specturm	Bandwidth	68.2 THz	5.3 THz	-	∼15 THz	-	55.1 THz
	Resolution bandwidth	625 MHz	20 MHz	-	5 MHz	-	3 MHz
	Dynamic range	65 dB	50 dB	-	40 dB	-	56 dB

lower branch. During the measurement, the laser chirps at 50 nm/s. The temporal beatnote signal is then directly recorded and transformed to the frequency domain with windowed fast Fourier transformation (FFT). The width of the FFT window T_w is inversely proportional to the FFT's frequency resolution δf as $\delta f = 1/T_\mathrm{w}$, which should be one order smaller than 99 kHz. In the experiment, we select $T_\mathrm{w} = 100$ μs, which corresponds to an FFT's frequency resolution δf of 10 kHz. We measured 12207 100-μs windowed beatnote signals' linewidth, the histogram is shown in Fig. S5c. The statistical laser linewidth corresponding to 100-μs is 471 kHz with a standard deviation of 292 kHz. Such a measured laser linewidth originates from multiple reasons, including the laser intrinsic linewidth, the laser chirp nonlinearity, and the instability of the fiber delay. It is hard to extract the linewidth induced by the laser intrinsic linewidth, thus we conclude 471 kHz as the frequency resolution of our VSA. Additionally, we also measure the frequency noise of the laser for reference. The laser is set to a specific frequency (i.e. 193.41449 THz) and combined with a fiber laser (NKT Koheras), the beatnote signal is directly analyzed with a phase noise analyzer (Rohde & Schwarz FSWP) The result is shown in Fig. S5b.

However, for spectrum detection, digital FIR filters of megahertz bandwidth are utilized. In this situation, to evaluate the VSA's performance, the resolution bandwidth is a more suitable parameter, which is the minimum spectral separation the spectrum analyzer can distinguish. In our experiment, the pass band 3-dB width of the FIR filter is designed to be 3 MHz, as shown in Fig. S5e, thus we infer 3 MHz as the spectrum measurement resolution bandwidth of our VSA. We further verify the result experimentally, the setup is shown in Fig. S5d, where the chirping laser sweeps across the reference laser (NKT Koheras) with a chirp rate of 50 nm/s. The reference laser is phase modulated with a 3 MHz sinusoidal signal to create sidebands. As shown in Fig. S5f, the sidebands can be unambiguously distinguished.

We finally compare the performance of our VSA with other commercial products and research advances, which is shown in Table S2. Our VSA provides a new paradigm to characterize integrated nonlinear devices over an ultra-wide bandwidth with a globally high resolution. Meanwhile, the VSA also provides comparable spectrum analyzing ability as the most advanced spectrum analyzer.

Supplementary Note 6. OVNA phase measurement

The setup of our VSA as an OVNA is shown in Fig. 3a in the main text. The branch containing a reference laser is for absolute frequency calibration. The branch containing the fiber cavity is for relative frequency calibration. Parallel to the branch where the laser transmits through the DUT, the laser passes through a 20-meter-long fiber, which introduces a time delay $\Delta\tau$. At the DUT's output, half of the laser is photodetected to generate the DUT's transmission trace. The other half interferes with the laser through the long fiber. In the following, we elaborate on how the phase information is extracted from the interference signal.

If the laser chirps at the rate of γ, there is a frequency difference $\gamma\Delta\tau$ between the two branches due to the long fiber, and a beat signal is generated. Mathematically, the chirping laser from the long-fiber branch can be formulated as

$$E_\mathrm{upper} \propto \exp[i\omega_0(t-\Delta\tau) + i\gamma(t-\Delta\tau)^2/2].$$

Similarly, for the DUT branch

$$E_\mathrm{lower} \propto \exp[i\omega_0 t + i\gamma t^2/2 + i\varphi(t)],$$

where $\varphi(t)$ is the phase shift introduced by the DUT. Therefore, the balanced photodetector output can be written as

$$I(t) \propto \mathrm{Re}(E_\mathrm{upper}E^*_\mathrm{lower}) = A\cos(\gamma\Delta\tau\cdot t + \varphi(t)),$$

where the constant phase $\omega_0\Delta\tau$ is merged into $\varphi(t)$, which can be canceled by introducing an additional reference path, and the high-order terms of $\Delta\tau$ are omitted. To extract the phase information $\varphi(t)$, Hilbert transformation is performed. In our case $\mathcal{H}[I(t)] = -A\sin(\gamma\Delta\tau\cdot t+\varphi(t))$, thus we have

$$\Phi(t) = \gamma\Delta\tau\cdot t+\varphi(t) = -\arctan\frac{\mathcal{H}[I(t)]}{I(t)}. \tag{S8}$$

In our experiment, $\gamma\Delta\tau$ is obtained by linear fit of $\Phi(t)$ far from the resonances. The phase $\varphi(t)$ can be further extracted with $\Phi(t)-\gamma\Delta\tau\cdot t$. With the absolute and relative frequency calibration, $\varphi(t)$ is then transformed from the time domain to the optical frequency domain, and the phase response of the DUT $\varphi(f)$ is finally obtained.

To illustrate the phase measurement process more explicitly, an example is shown in Fig. S6a. The red line shows the resonance's transmission within the laser chirping period, while the blue line is the beatnote recorded with the oscilloscope. The dashed black line is an amplitude-modulated sinusoidal curve as a phase reference, the phase is aligned with the beatnote far from the resonance. The additional phase $\varphi(t)$ introduced by the cavity's resonance shifts the phase of the beatnote by the same amount. In Fig. S6a, the phase of the blue line is shifted by $\varphi(t)$ near 60 MHz, and deviates from the dashed black line. The above-mentioned method is to extract the phase shift $\varphi(t)$ from the recorded time-domain beatnote.

With our OVNA, the microresonator's linewidth is characterized and shown in Fig. S6b. The resonance frequency $\omega/2\pi$ and linewidth of each fundamental-mode resonance, ranging from 1260 nm (237.9 THz) to 1640 nm (182.8 THz), are measured. For each resonance, the intrinsic loss $\kappa_0/2\pi$ and external coupling strength $\kappa_{\text{ex}}/2\pi$ are extracted by Lorentzian fit. With our phase measurement of resonances, we can unambiguously determine whether the resonance is over-coupled ($\kappa_{\text{ex}}>\kappa_0$) or under-coupled ($\kappa_{\text{ex}}<\kappa_0$).

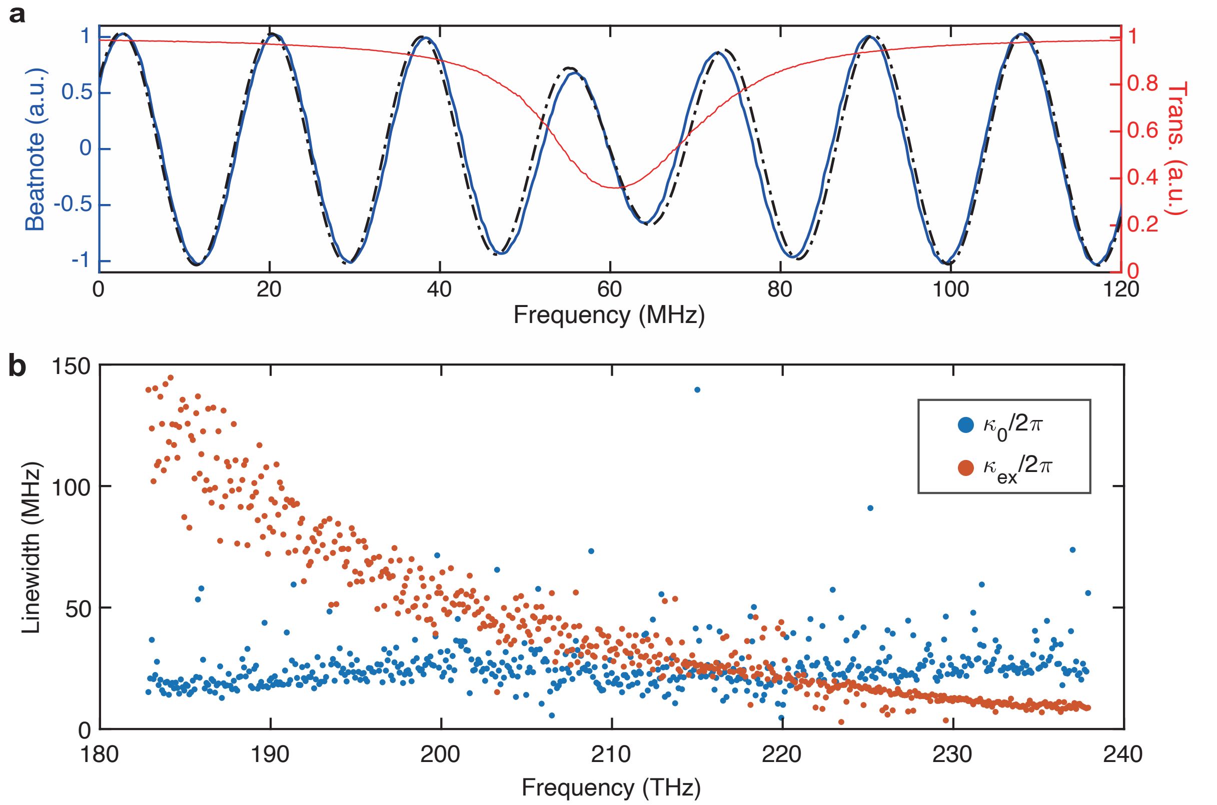

Supplementary Figure S6: Phase measurement. **a**. The beatnote recorded with the oscilloscope during the phase measurement process (blue line) and the resonance's transmission (red line). The dashed black line is an amplitude-modulated sinusoidal curve as a phase reference, the phase is aligned with the beatnote signal far from the resonance. The blue line deviates from the dashed black line due to the additional phase $\varphi(t)$ introduced by the resonance (around 60 MHz). **b**. Measurement of resonance linewidth over the 55.1 THz spectral bandwidth. The phase measurement allows unambiguous determination of whether the resonance is over-coupled ($\kappa_{\text{ex}}>\kappa_0$) or under-coupled ($\kappa_{\text{ex}}<\kappa_0$).

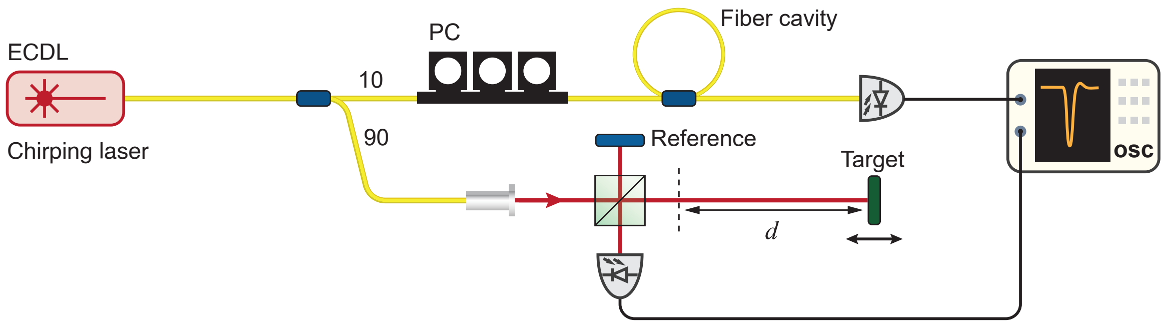

Supplementary Figure S7: Experimental setup of coherent LiDAR.

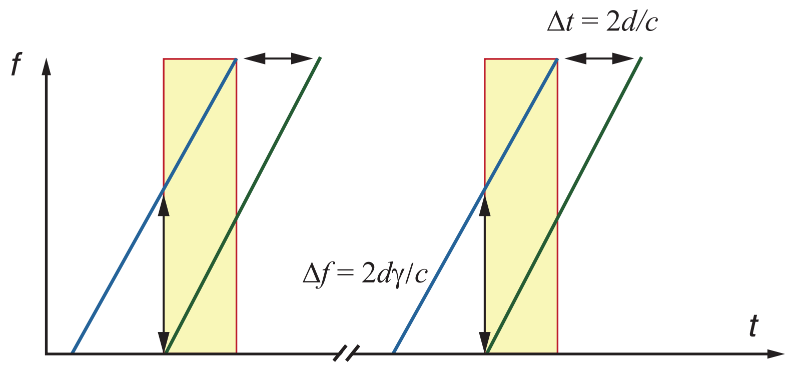

Supplementary Figure S8: Principle of coherent LiDAR.

Supplementary Note 7. Coherent LiDAR experiment

The experimental setup of coherent LiDAR is shown in Fig. S7. We use an ECDL that chirps from 192.2 THz to 194.7 THz. The duration is $T = 0.4$ s, thus the estimated linear chirp rate is $\gamma = 6.25$ THz/s. A 90:10 coupler is used to split the laser light into two parts. One part is directed to the VSA for frequency calibration, and the other is directed to a collimator and sent into free space.

In the free space, the chirping laser is split into two paths with path difference d and then recombined to interfere on a photodetector (FEMTO, HBPR-450M). As shown in Fig. S8, the path difference d introduces a time delay $\Delta t = 2d/c$, which generates a beat signal with frequency of $\Delta f = \gamma(t)\Delta t$, where $\gamma(t)$ is the time-dependent chirp rate. Thus, the beat signal can be written as:

$$\begin{aligned} V(t) &\propto \cos(2\pi\Delta f t) \\ &= \cos\left(2\pi\frac{2d}{c}\gamma(t)t\right) \end{aligned} \tag{S9}$$

From Eq. S9 we can see that the beat signal's frequency is $\Delta f = 2d \cdot \gamma(t)/c$, which can be obtained with fast Fourier transformation (FFT). In the ideal case where the chirp rate is constant, the path difference d can be directly extracted from the obtained Δf. In reality, the laser does not chirp linearly, i.e. the chirp rate $\gamma(t)$ fluctuates. Thus to extract d from Δf, an accurate trace of $\gamma(t)$ is required.

Fortunately, our VSA allows the monitor and record of the instantaneous laser frequency during its chirping. With known instantaneous laser frequency, the chirp rate $\gamma(t)$ can be calculated. Thus with calibrated $\gamma(t)$ we can re-scale the signal's time axis by $t' = \gamma(t)t$ to

$$V(t') = \cos\left(2\pi\frac{2d}{c}t'\right). \tag{S10}$$

Then the precise range profile can be retrieved.

In addition to chirp rate calibration, zero-padding[3] is also implemented in data processing, which inserts zeros before and after the measured data. As mentioned in the main text, the ranging resolution δd is limited by chirp bandwidth B as $\delta d = c/2B$ due to the constraints of the FFT. Zero-padding increases the bandwidth, which is useful for resolving ambiguities and reducing the quantization error in estimating the spectral peaks[3]. Despite this, zero padding can introduce additional noise through spectral leakage[4]. Given our broad measurement bandwidth and high precision of the relative frequency calibration, the beat signal exhibits a high signal-to-noise ratio (SNR). This high SNR enables accurate peak detection using 8192-fold zero-padding. It should be noted that zero-padding cannot improve the ranging resolution, i.e. resolving two objects distant less than $\delta d = c/2B$.

Supplementary References

[1] B. Shi, Y.-H. Luo, W. Sun, Y. Hu, J. Long, X. Bai, A. Wang, and J. Liu, arXiv , 2308.15875 (2023).
[2] T. Qing, S. Li, Z. Tang, B. Gao, and S. Pan, Nature Communications **10**, 5135 (2019).
[3] K. A. Shinpaugh, R. L. Simpson, A. L. Wicks, S. M. Ha, and J. L. Fleming, Experiments in Fluids **12**, 319 (1992).
[4] F. Zhang, Z. Geng, and W. Yuan, IEEE Transactions on Power Delivery **16**, 160 (2001).
